# ROS-responsive graphene-hyaluronic acid nanomedicine for targeted therapy in renal ischemia/reperfusion injury

**DOI:** 10.7150/thno.120560

**Published:** 2026-01-01

**Authors:** Seungjun Lee, Sang Heon Suh, Seong Kwon Ma, Junghyun Kim, Sehyeon Park, Soo Wan Kim, Jae Young Lee

**Affiliations:** 1 School of Materials Science and Engineering, Gwangju Institute of Science and Technology (GIST), Gwangju, 61005, Republic of Korea.; 2 Department of Internal Medicine, Chonnam National University Medical School, Gwangju, 61469, Republic of Korea.

**Keywords:** acute kidney injury, reactive oxygen species, graphene oxide, hyaluronic acid, paricalcitol

## Abstract

**Background**: Acute kidney injury (AKI) frequently progresses to chronic kidney disease (CKD) through the AKI-to-CKD transition; however, effective treatment strategies remain challenging due to the complex and multifactorial pathophysiology of this process. This study aims to develop a multifunctional nanoplatform for kidney-specific targeting, reactive oxygen species (ROS) scavenging, and anti-fibrotic drug delivery to mitigate AKI-to-CKD progression.

**Methods**: Reduced graphene oxide (rGO) was conjugated with hyaluronic acid (HA) to form HA/rGO nanoparticles, enabling CD44-mediated renal targeting and ROS-responsive drug release. Paricalcitol, a hydrophobic anti-fibrotic agent, was loaded onto HA/rGO to form P/HA/rGO. The physicochemical characteristics, ROS-scavenging capacity, and oxidative stress-responsive drug release were evaluated. *In vitro* cytoprotection was assessed using HK-2 cells under oxidative stress. *In vivo* studies using ischemia/reperfusion (IR) injury mouse models assessed biodistribution, renal targeting, and therapeutic efficacy after systemic administration of P/HA/rGO.

**Results**: HA/rGO nanoparticles demonstrated potent antioxidant activity and significantly protected HK-2 cells from ROS-induced cytotoxicity. P/HA/rGO exhibited a high paricalcitol loading efficiency (93%) and released 26% of the drug over 30 days under oxidative conditions. P/HA/rGO selectively accumulated in IR-injured kidneys via HA-CD44 interactions, decreased serum NGAL and cystatin C levels, and effectively attenuated tubular injury, fibrosis, inflammation, and apoptosis compared to vehicle-treated controls.

**Conclusion**: The P/HA/rGO nanoplatform enables kidney-targeted delivery of paricalcitol with ROS-scavenging and ROS-responsive release properties, providing a promising therapeutic strategy to suppress the AKI-to-CKD transition via integrated targeting and microenvironment-responsive therapy.

## Introduction

Acute kidney injury (AKI), characterized by a sudden decline in kidney function, is caused by a variety of etiologies, including ischemia, sepsis, and toxins 1. A major clinical concern is the frequent progression of AKI to chronic kidney disease (CKD) and end-stage kidney disease (ESKD) through the AKI-to-CKD transition 2, 3. Unfortunately, current treatments for AKI, which mainly rely on conservative modalities, remain ineffective due to the complex and multifactorial pathophysiology of AKI 1, 4. This pathophysiologic transition involves the generation of excessive reactive oxygen species (ROS) during the initial phase after ischemia/reperfusion (IR) injury, which triggers apoptotic and inflammatory processes in the tubulointerstitial compartment 5-7. In the absence of specific treatments, interstitial fibroblasts are activated during the late phase after IR injury, ultimately leading to renal fibrosis 8-10. Consequently, the development of innovative therapeutics that can neutralize ROS and inhibit fibrosis is highly required to effectively mitigate the AKI-to-CKD transition.

**Scheme 1:**
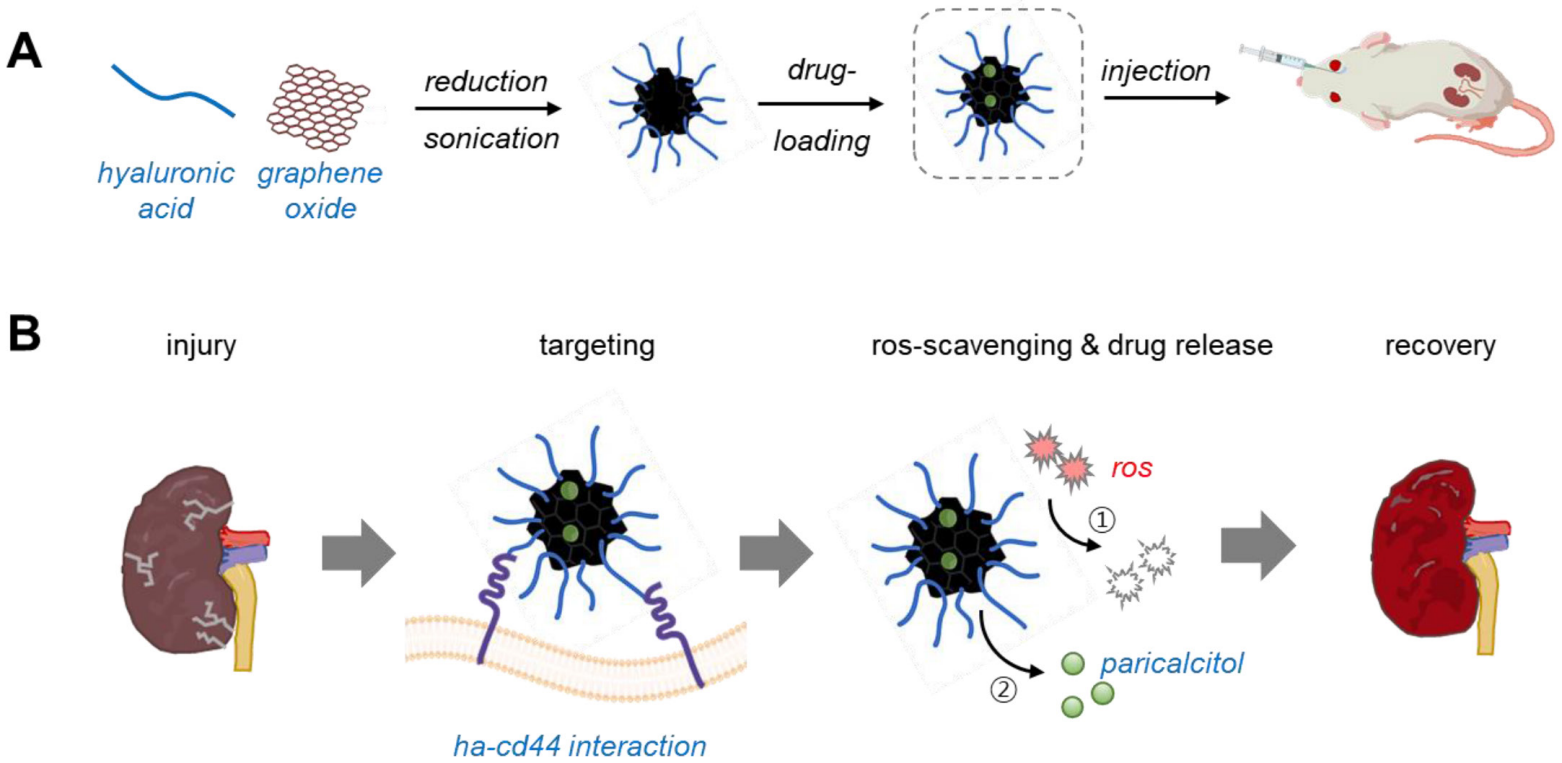
Schematic illustration of paricalcitol-loaded reduced hyaluronic acid-conjugated reduced graphene oxide nanoparticles (P/HA/rGO) for the treatment of ischemia-reperfusion (IR) renal injury. (A) Synthesis of P/HA/rGO via *in situ* reduction and high-power sonication, followed by paricalcitol loading and intravenous administration to acute kidney injury (AKI) mice. (B) Multifunctional therapeutic characteristics of P/HA/rGO, including specific targeting to injured kidney via HA-CD44 interactions, ROS scavenging, and ROS-responsive drug release, for suppressing pathological progression of AKI.

Concurrently, the precise delivery of therapeutics to injured kidneys under pathological conditions is essential to enhance therapeutic efficiency and minimize undesirable side effects. To this end, we developed a multifunctional nanoplatform capable of targeting injured kidneys, scavenging ROS, and locally releasing anti-fibrotic drugs for AKI treatment (Scheme 1). Reduced graphene oxide (rGO) was utilized as both an antioxidant and a carrier for hydrophobic molecules 11-13. Hyaluronic acid (HA) was conjugated to rGO to produce HA/rGO nanoparticles that allow for kidney-specific delivery via specific interactions with CD44, a ligand overexpressed in injured kidney tissues 14-16. The vitamin D receptor agonist paricalcitol (19-nor-1,25-dihydroxyvitamin D₂), as anti-fibrotic drug, was loaded onto HA/rGO to finally yield the multifunctional therapeutic formulation (P/HA/rGO) 17, 18. We characterized the physicochemical and biological properties of the synthesized HA/rGO and P/HA/rGO, including morphology, composition, antioxidant activity, ROS-responsive drug release, and cytocompatibility. Moreover, we performed *in vivo* experiments using an IR renal injury model to evaluate the therapeutic efficacy of intravenously injected HA/rGO for targeting and repairing injured kidneys. Importantly, we elaborated to evaluate the potential of P/HA/rGO as a therapeutic option to prevent the AKI-to-CKD transition in IR-induced AKI.

## Materials and methods

### Materials

A highly concentrated single-layer graphene oxide (GO) solution (6 mg/mL) was obtained from Graphene Supermarket, Inc. (Ronkonkoma, NY, USA). Sodium hyaluronate (Pharma Grade, molecular weight: 1,200-1,900 kDa) from Kikkoman Biochemifa Company (Tokyo, Japan). Phosphate buffered saline (PBS), L-ascorbic acid, 2,2-diphenyl-1-picrylhydrazyl (DPPH), 2,2ʹ-azino-bis (3-ethyl-benzthiazoline-6-sulfonic acid (ABTS), potassium persulfate (KPS), hydrogen peroxide (H_2_O_2_), Sudan Black B, Canada balsam sodium citrate, and citric acid were purchased from Sigma-Aldrich (St Louis, MO, USA). Minimum Essential Medium (MEM)-alpha, fetal bovine serum (FBS), antibiotic-antimycotic (100 ×; penicillin 10,000 unit/mL, streptomycin 10,000 μg/mL, and amphotericin 25 μg/mL), Dulbecco's phosphate-buffered saline (DPBS), trypsin/EDTA (0.05%), Dulbecco's Modified Eagle Medium/Nutrient Mixture F-12 (DMEM/F12), goat serum, HPLC grade methanol, HPLC grade acetonitrile, and Live/Dead ^TM^ viability/cytotoxicity kit were purchased from Thermo Fisher Scientific (Waltham, MA, USA). The dialysis membrane (12-14 kDa) was purchased from Spectrum Laboratories (Rancho Dominguez, CA, USA). Lactate dehydrogenase (LDH) Cytotoxicity Detection Kit was purchased from Takara Shuzo Co. (Otsu, Shiga, Japan). Ethanol was purchased from DUKSAN Pure Chemicals (Incheon, Republic of Korea). Cyanine 5 amine was purchased from Lumiprobe (Wan Chai, Hong Kong). Paricalcitol was purchased from AdooQ^®^ bioscience (Irvine, CA, USA).

### Preparation and characterization of HA-conjugated rGO nanoparticles

#### Synthesis of HA/rGO

The GO solution (6 mg/mL, 3 mL) was diluted with deionized water 15 mL of (DI) to achieve a final concentration (1 mg/mL). HA was completely dissolved in the GO solution at a concentration of 0.05 wt%. The HA/GO solution was subjected to tip sonication (VC750, Sonics & Materials Inc., Newtown, CT, USA) for 30 min at 22% amplitude with an on/off cycle of 8/7 s. During sonication, 100 mg of ascorbic acid was added dropwise to 10 mL of the GO/HA solution for *in situ* reduction. Finally, the solution was dialyzed using a dialysis membrane (molecular weight cut-off: 1,000 kDa) in DI water for 3 days to remove unbound HA and residual ascorbic acid. The resultant HA/rGO was stored at 4 °C till use. As a control, HA/GO was prepared in a manner identical to HA/rGO, except for the addition of ascorbic acid.

#### Physicochemical characterization of HA/rGO

To determine hydrodynamic size and zeta potential, 10 μL of each sample solution was diluted with 990 μL of DI water and analyzed at 37 °C using a zeta potential and particle size analyzer (ELSZ-2000, Otsuka electronics, Osaka, Japan). *In vitro* stability of GO, rGO, and HA/rGO were assessed by visually monitoring their aggregation or sedimentation in solution during incubation. Each diluted solution (0.2 mL) was mixed with growth medium (1.8 mL; MEM-alpha supplemented with 10% FBS and 1% antibiotic-antimycotic) and incubated at 37 °C for 7 days. The morphologies of GO and HA/rGO were analyzed using transmission electron microscopy (TEM; Tecnai G2 F30 S-Twin System, FEI Company, Hillsboro, OR, USA). The TEM sample was prepared by drop casting the sample solution onto a carbon-coated grid. The reduction of GO flakes in the various samples was analyzed using Raman spectroscopy. Briefly, samples were drop-casted onto clean glass slides and dried overnight in a desiccator. Raman spectra were obtained using a Raman spectroscope (LabRAM HR Evolution, Horiba Scientific, Piscataway, NJ, USA) equipped with a 532 nm laser. D and G band intensities were measured, and the intensity ratio (I_D_/I_G_) in each spectrum was calculated with WiRE 3.2 software (RENISHAW, Gloucestershire, UK) (*n*=5). Fourier-transform infrared (FTIR) spectroscopy and thermogravimetric analysis (TGA) were performed using freeze-dried samples (GO and HA/rGO). The FTIR spectrum was obtained using an FTIR spectroscope (Vertex 70 v, Bruker, Boston, MA, USA) in attenuated total reflection mode, with a peak resolution of 4 cm^-1^ in the range of 4,000-600 cm^-1^ and 64 scans per sample. TGA was performed using TGA-50 (SHIMADZU, Kyoto, Japan) at a heating rate of 10 °C/min from 10 to 600 °C in a nitrogen atmosphere to quantify the composition of GO and HA in the nanoparticles.

### Antioxidant ability tests

Antioxidant activity was evaluated using DPPH and ABST assays. For the DPPH assay, DPPH was dissolved in 80% (v/v) methanol to prepare a 0.1 mM stock solution. The stock solution was further diluted with 80% methanol to prepare a DPPH assay solution with an absorbance of 0.70 ± 0.02 at 517 nm. Each sample (100 μg) was dissolved in 900 μL of the DPPH assay solution and further incubated at 25 °C for 1 h. Absorbance of the supernatant was measured at 517 nm using a plate reader (SpectraMax M2, San Jose, CA, USA). NaCl solution (0.15 M) was used as a control. The scavenging activities of the samples were calculated using the following equation (1):


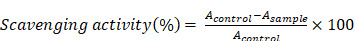
(1)

For the ABTS assay, a stock solution containing 7.4 mM ABTS and 2.6 mM KPS was prepared in PBS. The stock solution was then diluted with PBS to prepare an ABST assay solution with an absorbance of 0.70 ± 0.02 at 732 nm. Each sample (100 μg) was dissolved with 900 μL of the ABTS assay solution and incubated at 25 °C for 1 h. Absorbance of the supernatant was measured at 732 nm using a microplate reader. NaCl solution (0.15 M) was used as a control. The scavenging activity was determined according to Equation (1), in the same manner as applied for the DPPH assay.

### Drug loading and release tests

#### Drug loading

Paricalcitol was dissolved in 100% ethanol at 10 mg/L and further diluted with 100% ethanol to obtain a final concentration of 5 mg/L. Then, 3 mL of the HA/rGO solution containing GO was mixed with 2 mL of the drug stock solution. The mixture was incubated in a shaking incubator at 50 rpm and 37 °C for 48 h for drug adsorption. The concentration of paricalcitol in the solution after incubation was quantified using a high-performance liquid chromatograph (HPLC; Agilent HPLC Infinite 3000, Santa Clara, CA, USA) equipped with a UV detector to measure the absorbance at 250 nm. HPLC was performed using a mobile phase (a mixture of acetonitrile and water at a 70:30 (v/v) ratio) at a flow rate of 1 mL/min. A series of standard solutions were prepared by diluting the drug stock solution to a concentration range of 0.1-7.5 mg/L. A standard paricalcitol curve was obtained with peak areas (*A*) and paricalcitol concentrations (*C*), which had a linear relationship in a concentration range of 0-7.5 mg/L with a regression equation of A = 7.4067C with R² = 0.999. The equilibrium adsorption efficiency (*Ee* [%]) and capacity (*Qe* [μg/g]) were calculated by using the following equations (2) and (3) 19.


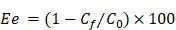
(2)


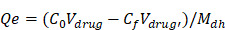
(3)

where *C_0_* is an initial paricalcitol concentration, *C_f_* is a paricalcitol concentration in the solution after incubation with HA/rGO, *V_drug_* is an initial volume of the paricalcitol solution, *V_drug_*_'_ is a volume of the paricalcitol solution after incubation with HA/rGO, and *M_dh_* is weight of GO.

#### Drug release tests

After paricalcitol adsorption, the P/HA/rGO nanoparticles were thoroughly washed to remove the unloaded paricalcitol prior to the release tests. In brief, P/HA/rGO was suspended in 40% ethanol and dialyzed using a dialysis tube (molecular weight cut-off: 3.5 kDa) in 30% ethanol solution at 25 °C with a gentle stirring (200 rpm), followed by sequential dialysis in 20% ethanol for 1 h and 10% ethanol for 1 h, and finally in DI water for 21 h. Then, the P/HA/rGO was collected from the dialysis tube and used for the subsequent drug release tests. P/HA/rGO (1 mg) was incubated in 1 mL of release buffer (0.05% (v/v) Tween 20 in 1× PBS) in a sealed Eppendorf tube at 37 °C with shaking at 200 rpm. For drug release under non-oxidative conditions, P/HA/rGO was incubated in the release buffer for 30 days. For drug release tests under oxidative stress, P/HA/rGO was incubated in a release buffer containing 1 mM H_2_O_2_ for 30 days. The supernatant from each sample was collected on days 1, 5, 12, 20, and 30, and centrifuged at 15,000 rpm for 20 min. The concentration of paricalcitol in the supernatant was analyzed using HPLC as described previously.

### *In vitro* cell culture studies

#### Cell culture

HK-2 cells (an immortalized human kidney proximal tubular epithelial cell line) and NRK49F cells (a rat kidney interstitial fibroblast cell line) were obtained from American Type Culture Collection (Manassas, VA, USA). HK-2 cells serve as a model of proximal tubular epithelium, and NRK49F cells are used as a representative of renal fibroblasts 20. HK-2 cells were cultured in DMEM/F-12 supplemented with 10% FBS, 100 U/mL penicillin, and 100 mg/mL streptomycin. NRK49F cells were maintained in DMEM/high glucose media (WelGene, Daegu, Republic of Korea) supplemented with 5% FBS, 50 U/mL penicillin, and 50 μg/mL streptomycin. Cells were maintained in a humidified incubator with 5% CO_2_ at 37 °C for and sub-cultured at 70-80% confluence. For the assessment of the direct inhibitory effect of P/HA/rGO on the fibroblast activation, NRF49F cells were treated with 50 μg/mL P/HA/rGO for 1 h and subsequently with 5 ng/mL TGF-β1 (Sigma-Aldrich) for 24 h.

#### Cytocompatibility tests

Live/dead staining was performed according to the manufacturer's instruction (Thermo Fisher Scientific). Briefly, the staining solution was prepared by mixing 20 μL of EthD-1 (2 mM), 5 μL of calcein AM (5 mM), and 10 mL of DPBS. The staining solution was added to HK2 cells and incubated at 37 °C in a humified incubator with 5% CO₂ at 37 °C for additional 10 min. Subsequently, cells were washed twice in DPBS (5 min per wash), and fluorescence images were obtained using a fluorescence microscope (Leica DMI3000 B, Wetzlar, Germany). Green (live) and red (dead) cells were quantified from randomly selected fluorescence images (*n*=5). Cell viability was determined according to Equation (4):


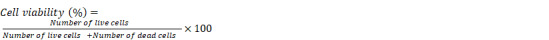
 (4)

The cytocompatibility was further examined using an LDH assay. HK2 cells were incubated with HA/rGO nanoparticles at various concentrations (5, 10, 50, or 100 μg/mL) for 24 h, and the culture medium was collected for analysis. The medium from cells cultured on a tissue culture plate was used as the low control (live cells), whereas the medium from cells treated with 2% Triton X-100 was used as the high control (dead cells). Each culture medium was mixed with the assay reagent in a 1:1 volume ratio and incubated at 25 °C for an additional 30 min. The assay reagent was freshly prepared before use by mixing 250 μL of LDH catalyst solution and 11.25 mL of LDH dye solution. The absorbance of each sample was measured at 490 nm using a microplate reader. LDH levels were determined according to Equation (5):


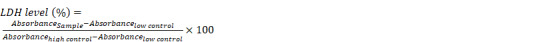
 (5)

The cytoprotective effects of rGO and HA/rGO against ROS were examined in HK2 cells. HK2 cells were incubated with 100 μg/mL of either rGO or HA/rGO at various concentrations in the culture medium containing 500 mM H_2_O_2_ for 24 h. Then, the culture medium was collected, and LDH assay was performed in an identical manner described above.

#### Cellular uptake test for HA/rGO and P/HA/rGO

HK2 cells were seeded in 6-well plates at a density of 5.0 × 10^5^ cells/well and incubated at 37 °C with 5% CO₂ for 24 h. The cells were then treated with 100 mM H_2_O_2_ for 2 h to induce oxidative stress, followed by incubation with 100 μg/mL of either rGO or HA/rGO for additional 6 h. The cells were washed with DPBS three times, fixed by incubation in 3.74% paraformaldehyde solution at 25 °C for 20 min, and washed with DPBS. Dark-field images of nanoparticle scattering in intracellular regions were acquired using a microscope (Leica DMI 3000 B, Leica Microsystems Ltd.) equipped with a dark-field condenser (Leica P 1.40 Oil S1, Leica Microsystems Ltd.).

The intracellular paricalcitol levels in cells treated with P/HA/rGO were quantified. In brief, HK2 cells were seeded in 6-well plate at a density of 5.0 × 10^5^ cells/well and incubated in the growth medium for 24 h. Then, the cells were treated with 100 mM H_2_O_2_ for 2 h and further incubated with either paricalcitol (0.93 μg/mL) or P/HA/rGO (1 mg/mL containing 0.93 μg/mL paricalcitol) for 6 h. After treatment, cells were washed twice with DPBS and detached by incubation in 0.05% trypsin/EDTA solution (1 mL) at 37 °C for 10 min, followed by addition of growth medium (2 mL) for neutralization. The cell suspension was centrifuged at 15,000 rpm for 15 min, and the obtained cell pellet was incubated in 1 mL of TRIzol at 25 °C for 5 min. Then, 0.2 mL of chloroform was added, followed by vigorous mixing for 15 s and incubation for 3 min at 25 °C. The samples were centrifuged at 15,000 rpm for 15 min at 4 °C. The organic phase was collected and carefully evaporated under nitrogen stream. The residue was dissolved in 40% ethanol, and the extracted paricalcitol was quantified using HPLC as described above.

### *In vivo* animal studies

#### Animal model establishment and surgical procedures

All experimental procedures were reviewed and approved by the Animal Care Regulations Committee of the Chonnam National University Medical School CNUHIACUC-24035). C57BL/6J wild-type (WT) and *Cd44*^-/-^ mice (Jackson stock number 0050859) were purchased from Jackson Laboratory (Bar Harbor, ME, USA). A unilateral IR renal injury model was used to induce renal fibrosis as previously described 21. To assess the therapeutic efficacy of P/HA/rGO in IR renal injury, experimental mice were classified into three groups: *i*) sham-operated mice with vehicle injection (Sham-Vehicle), *ii*) IR-injured mice with vehicle injection (IR-Vehicle), and *iii*) IR-injured mice with P/HA/rGO injection (IR-P/HA/rGO). Eight-week-old male mice were anesthetized by an intraperitoneal injection of a mixture of ketamine and xylazine. After removal of skin hair and completion of antiseptic procedures, the right kidney was exposed by incising the skin and muscle layers. Ischemia was induced by clamping the right renal pedicle for 25 min. Perfusion was allowed at the end of ischemic insult. Sham surgery was performed using an identical surgical procedure, except for clamping. Finally, 100 μL of P/HA/rGO (0.32 mg/kg of P/HA/rGO, delivering 0.3 μg/kg of paricalcitol in a mouse) or vehicle (*i.e.*, PBS) was intravenously delivered via the retro-orbital route upon the visual confirmation of the reperfusion (*i.e.*, the recovery of the normal kidney color).

### Biodistribution of P/HA/rGO in IR renal injury mice

Fluorescence dye-incorporated P/HA/rGO nanoparticles were prepared using Cy5-conjugated HA for visual evaluation of their *in vivo* distribution. Cy5-labeled HA was first synthesized using the EDC/NHS chemistry. In brief, HA (1 MDa) was dissolved in MES buffer (0.1 M, pH 4.5) at a concentration of 0.125 mM, and NHS and EDC were dissolved in MES buffer at concentrations of 0.25 and 0.375 mM, respectively. The EDC/NHS solution was added to the HA solution and incubated under stirring at 200 rpm for 15 min. Cy5 (0.625 mM in MES) was then added dropwise to the reaction solution and allowed to react for 24 h. The reaction mixture was dialyzed in DI water for three days while a daily exchange of DI water and finally lyophilized. Paricalcitol was loaded into the fluorescence-labeled HA/rGO as previously described. P/HA/rGO was intravenously injected at the end of the ischemic or sham operation. Mice were sacrificed 48 h after IR injury. The harvested organs were analyzed using a fluorescence optical bioimaging system (FOBI, Cellgentek Co., Daejeon, Republic of Korea) to determine the biodistribution of P/HA/rGO nanoparticles. The fluorescence intensity of each organ was measured using NEO Image (Ver 3.3) FOBI software with 3000 ms exposure and 5× gain.

### Histological and immunohistochemical analyses

The harvested organs were fixed with 4% paraformaldehyde (in PBS) by cardiac perfusion. Paraffin blocks were prepared for histochemical staining as previously described 22. Briefly, the harvested organs were post-fixed in 10% neutral-buffered formalin (Sigma-Aldrich) at 25 °C overnight and embedded in paraffin. The paraffin blocks were sectioned to 3-μm thick slices, which were used for subsequent hematoxylin and eosin, periodic acid-Schiff, and Picrosirius red staining 22.

For immunohistochemistry, frozen kidney sections were further processed following cardiac perfusion, as previously described 23 with minor modifications. The kidneys were post-fixed with 4% paraformaldehyde in ice-cold PBS for 2 h and dehydrated with 20% sucrose (in PBS) at 4 °C overnight, followed by embedding in optimal cutting temperature compound and sectioning to 20-µm thick slices. After rehydration with PBS, the sections were incubated with the primary antibodies diluted in blocking buffer (1% [w/v] bovine serum albumin dissolved in 0.3% [v/v] Triton X-100 [in PBS]) at 4 °C overnight. Sections were briefly washed with 0.3% Triton X-100 solution (in PBS) and incubated with secondary antibodies at 25 °C for 1 h. The samples were washed thrice with 0.3% Triton X-100, once with PBS, and stained with DAPI. Fluorescence images were acquired using a confocal microscope (LSM 800; Carl Zeiss, Oberkochen, Germany). Information on the primary and secondary antibodies employed for immunohistochemistry is described in Table S1.

### Determination of serum neutrophil gelatinase-associated lipocalin (NGAL) and cystatin C levels

Whole blood was centrifuged at 2,000 × *g* for 10 min to collect the blood supernatant. NGAL and cystatin levels in the blood supernatant were quantified using ELISA kits (R&D Systems, Minneapolis, MN, USA) according to the manufacturer's instructions.

### Immunoblotting

For the semi-quantitative analysis of proteins in the kidney after surgery and treatment, immunoblotting was performed according to a previously described method 24. Kidney homogenates or cell lysates were prepared using modified radioimmunoprecipitation assay buffer (RIPA buffer; 150 mM sodium chloride, 50 mM Tris-HCl [pH 7.4], 1 mM EDTA, 1% v/v Triton X-100, 1% w/v sodium deoxycholic acid, 0.1% v/v sodium dodecyl sulfate [SDS]) and further centrifuged at 4,000 × *g* for 15 min at 4 °C. The total protein content in each sample was quantified using a bicinchoninic acid assay kit (Pierce, Rockford, IL, USA), and all samples were adjusted to equal concentrations. The supernatant was incubated in loading buffer at boiling temperature for 5 min to prepare the loading samples. The samples were loaded onto 6-12% SDS-polyacrylamide gel electrophoresis gels and electro-transferred onto nitrocellulose membranes (Hybond ECL RPN3032D; Amersham Pharmacia Biotech, Little Chalfont, UK) using a Bio-Rad Mini Protean II apparatus (Bio-Rad, Hercules, CA, USA). The membranes were incubated in the blocking buffer (80 mM Na_2_HPO_4_, 20 mM NaH_2_PO_4_, 100 mM NaCl, and 0.1% Tween-20 at pH 7.5) at 25 °C for 1 h, and further incubated with primary antibodies at 4 °C overnight. Membranes were briefly washed and incubated with secondary antibodies at 25 °C for 1 h. Immunoblots were developed using an enhanced chemiluminescence kit (EMD Millipore). Densitometric quantification was conducted using Scion Image software (Scion Corp, Frederick, MD, USA). Information on the primary and secondary antibodies applied for immunoblotting is described in Table S2.

### Quantitative reverse transcription polymerase chain reaction (RT-qPCR)

RT-qPCR of kidney tissues after surgery and treatment was performed according to a previously described method 20. Briefly, the kidneys were snap-frozen and homogenized in TRIzol reagent (Invitrogen, Carlsbad, CA, USA). Total RNA was extracted from tissue homogenates using the RNeasy Mini Kit (Qiagen, Mississauga, Canada). Then, 5 μg of the total RNA was reverse-transcribed to cDNA using SuperScript II Reverse Transcriptase (Invitrogen). Relative mRNA expression was analyzed by RT-qPCR with the Rotor-Gene 3000 Detector System (Corbette Research, Mortlake, New South Wales, Australia). Information on the primers used for qPCR is described in Table S3.

### Statistical analysis

All experiments were performed in triplicates unless stated otherwise. Statistical significance was calculated using one-way ANOVA with Tukey's multiple comparison test or by two-way ANOVA with Sidak's multiple comparisons test using Origin Pro 9.1 software. Differences were considered significant at *p* < 0.05.

## Results

### Synthesis and characterization of HA/rGO

**Figure 1:**
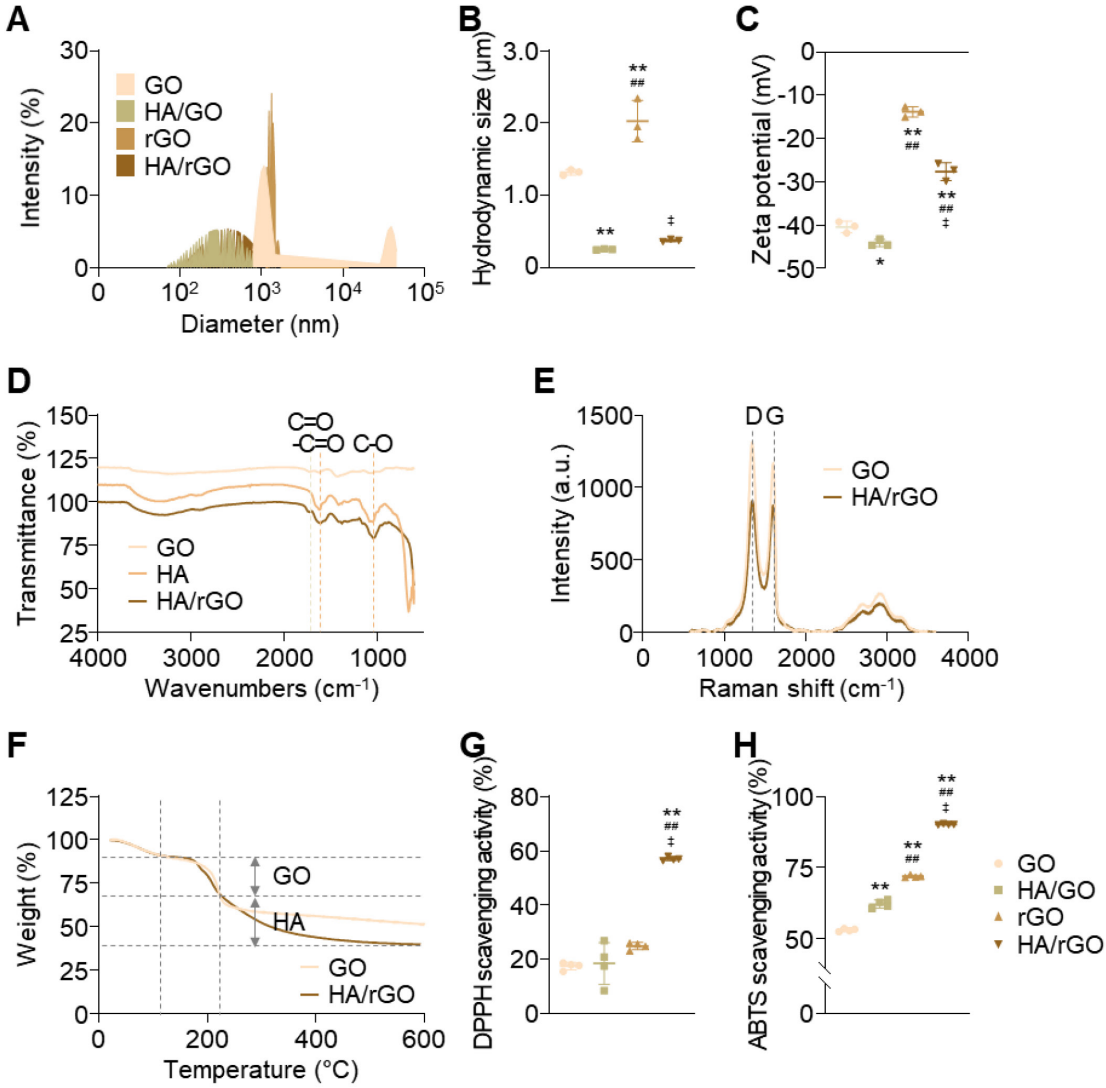
Physicochemical characterization of nanoparticles. Experimental nanoparticles include graphene oxide flakes (GO), hyaluronic acid (HA)-conjugated GO nanoparticles (HA/GO), reduced GO nanoparticles (rGO), and HA-conjugated rGO nanoparticles (HA/rGO). HA conjugation was performed by *in situ* high-power sonication. (A) Dynamic light scattering (DLS) size distributions. (B) Hydrodynamic sizes. (C) Zeta potentials. (D) Fourier-transform infrared spectra. (E) Raman spectra. (F) Thermogravimetric analysis (TGA) thermograms. (G) DPPH and (H) ABTS assays for anti-oxidizing activities evaluation. *P < 0.05, **P < 0.01 *vs.* GO; ^##^*P* < 0.01 *vs.* HA/GO; ^‡^*P* < 0.01 *vs.* HA/rGO.

HA/rGO nanoparticles were synthesized via the *in situ* chemical reduction of a solution containing HA and GO by adding ascorbic acid (10 mg/mL) while applying high-power sonication. High-power sonication significantly decreased the hydrodynamic size of GO flakes from 1,321 ± 31 nm to 256 ± 6 nm (Figure 1A-B). The resultant HA/rGO had a hydrodynamic size of 378 ± 13 nm, which was larger than that of the GO sonicated in the HA-free solution but still smaller than that of pristine GO. Notably, the rGO prepared by ascorbic acid treatment and high-power sonication without HA had an extremely large hydrodynamic size (2,026 ± 235 nm) due to agglomeration of hydrophobic rGO flakes in the absence of HA (Figure 1A-B). In contrast, the absence of severe aggregation of HA/rGO implied the possible incorporation of HA into the rGO flakes during fragmentation by high-power sonication and reduction, indicating enhanced colloidal stability. Scanning electron microscopy (SEM) and transmission electron microscopy (TEM) images indicated well-fragmented morphologies of HA/rGO without severe aggregation (Figure S1). A zeta potential of HA/rGO (-28 ± 2 mV) was less negative than that of pristine GO (- 40 ± 1 mV) and HA/GO (-44 ± 1 mV) but significantly more negative than that of rGO (-13 ± 1 mV), implying successful incorporation of HA moieties into rGO during reduction and sonication (Figure 1C).

The effects of the ascorbic acid concentration on the size and zeta potential of HA/rGO were investigated (Figure S2). At 1-10 mg/mL of ascorbic acid, the resulting HA/rGO nanoparticles exhibited similar hydrodynamic sizes (approximately 380 nm). However, the HA/rGO nanoparticles produced at 20 mg/mL of ascorbic acid exhibited a significantly larger size (482 ± 24 nm) than that of other nanoparticles, likely due to severe aggregation induced by hydrophobic interactions between rGO (Figure S2A). Interestingly, the zeta potential remained unaffected at ascorbic acid concentrations below 20 mg/mL, suggesting that ascorbic acid did not compromise HA immobilization onto the rGO surface (Figure S2B). Hence, 10 mg/mL of ascorbic acid was determined to be suitable for the production of well-dispersed rGO/HA nanoparticles and used in subsequent experiments. Note that the HA/rGO produced at this ascorbic acid concentration exhibited significantly improved antioxidant activity (Figure S3) and paricalcitol loading efficiency (Figure S4).

Fourier Transform Infrared (FTIR) spectroscopy further confirmed the successful incorporation of HA into HA/rGO. The spectrum of HA/rGO exhibited the characteristic peaks of GO at 1733 cm^-1^ (C=O stretching) and HA at 1627 cm^-1^ (amide I stretching) and 1045 cm^-1^ (C-O stretching) (Figure 1D). Raman spectroscopy was performed to evaluate the reduction of the GO component in HA/rGO by comparing the intensity ratios (*I_D_*/*I_G_*) of the D and G bands. The D band at 1350 cm^-1^ is associated with structural defects, whereas the G band at 1580 cm^-1^ indicates sp^2^ carbon atom vibrations 25. The *I_D_*/*I_G_* ratio of the GO components decreased from 1.24 ± 0.01 to 1.12 ± 0.01 after *in situ* sonication and reduction (Figure 1E), suggesting the restoration of the sp^2^ bonds and the reduction of defects in the GO flakes 26, 27. Thermogravimetric analysis (TGA) results indicated that the GO and HA contents of HA/rGO were 36.7 and 63.3%, respectively (Figure 1F).

The colloidal stability of GO, rGO, and HA/rGO was tested in serum-containing culture media at 37 °C (Figure S5A). HA-free rGO nanoparticles rapidly sedimented in 12 h and exhibited severe aggregation with an increase in hydrodynamic size to 3761 ± 259 nm and a polydispersity index (PDI) of 1.56 ± 0.25 (Figure S5B-C). The GO nanoparticles displayed only a slight increase in hydrodynamic size and remained stable without noticeable aggregation by 5 days. However, after 5 days of incubation, aggregation and sedimentation were observed with an increased hydrodynamic size to 2790 ± 138 nm and a PDI of 0.82 ± 0.04. In contrast, HA/rGO demonstrated excellent colloidal stability, maintaining its dispersion without significant aggregation or sedimentation for up to 7 days. The hydrodynamic size and PDI of HA/rGO remained consistently below 500 nm and 0.35, respectively, throughout the test period. Similarly, drug loaded HA/rGO exhibited good colloidal stability in more physiologically relevant fluids, specifically 50% mouse blood serum, (Figure S6). This enhanced stability can be attributed to the smaller particle size and the hydrophilic nature of HA/rGO, which effectively prevented aggregation and sedimentation.

As we aimed to develop a nanomedicine capable of effectively scavenging ROS and actively releasing drugs in response to ROS to treat IR renal injury, the ROS-scavenging activity of the nanoparticles was assessed using 2,2-diphenyl-1-picrylhydrazyl (DPPH) and 2,2′-azino-bis (3-ethylbenzothiazoline-6-sulfonic acid) (ABTS) assays (Figure 1G-H). While GO and HA/GO had moderate antioxidant activities, HA/rGO presented substantially improved antioxidant activity. HA/rGO exhibited 2.3- and 1.3-fold higher anti-oxidizing abilities than those of rGO in the DPPH and ABTS assays, respectively. These findings indicate that HA/rGO exhibits substantially enhanced antioxidant activity by *in situ* sonication and reduction, and is expected to effectively attenuate excessive oxidative stress after IR-induced renal injury.

### *In vitro* cytocompatibility and antioxidant effects of HA/rGO under oxidative stress

**Figure 2:**
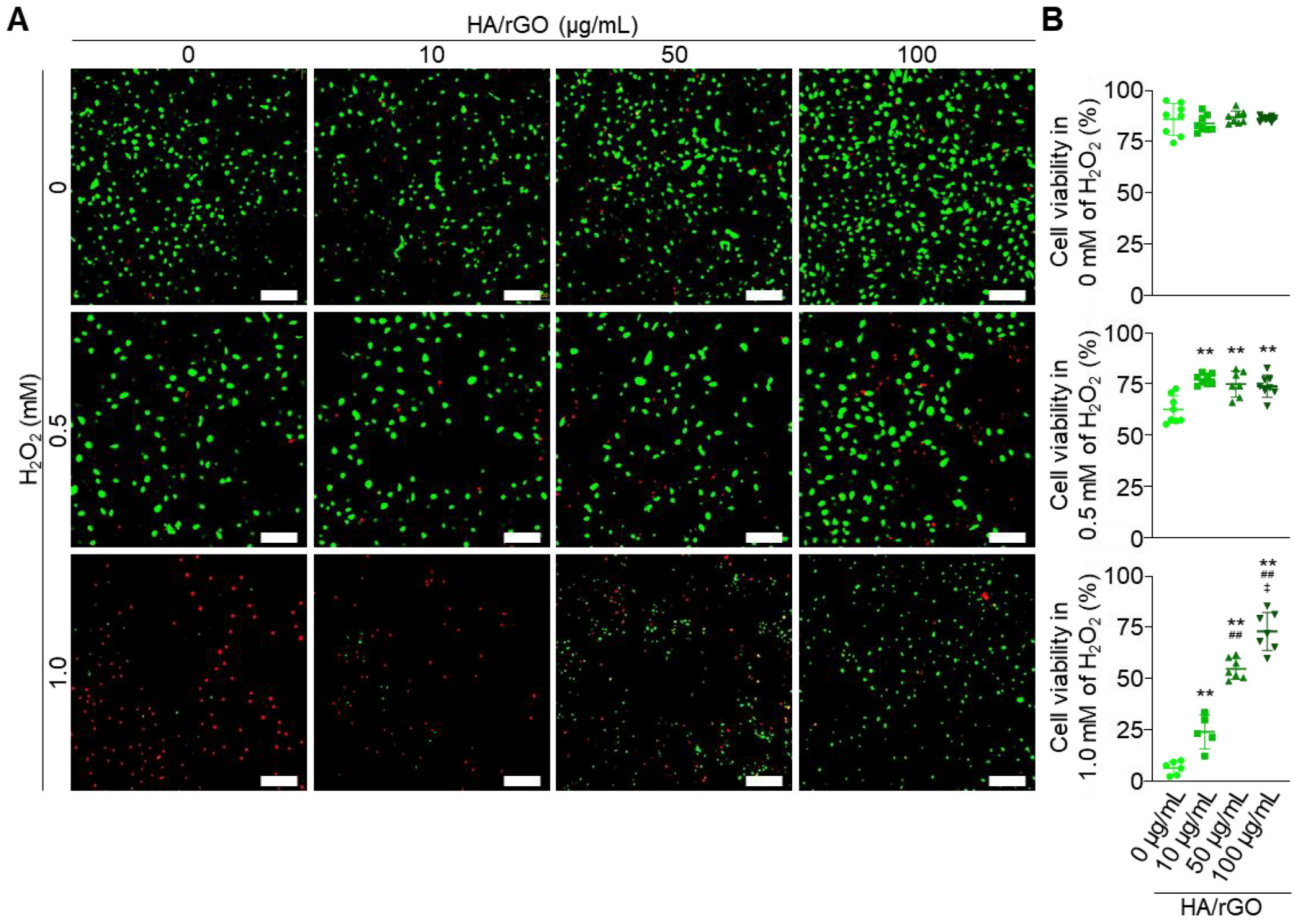
Viability of the HK2 cells under normal and oxidative stress conditions. (A) Live/dead fluorescence images and (B) cell viabilities measured after 24 h incubation at 0, 0.5, and 1 mM H_2_O_2_ with HA/rGO at various concentrations (0, 10, 50, and 100 μg/mL). Scale bars = 200 μm. **P < 0.01 *vs.* 10 μg/mL of HA/rGO; ^##^*P* < 0.01 *vs.* 50 μg/mL of HA/rGO; ^‡^*P* < 0.01 *vs.* 100 μg/mL of HA/rGO.

The cytocompatibility of the nanoparticles was first assessed under normal non-oxidative stress conditions using HK2 cells (Figure 2A). The HA/rGO exhibited excellent cytocompatibility with cell viability (> 80%) at the concentrations up to 100 μg/mL (Figure 2B). Cell viability was also assessed under oxidative stress induced by H_2_O_2_ (0.5 or 1 mM), in the presence or absence of HA/rGO treatment. At 0.5 mM H_2_O_2_, the cell viability was significantly higher in the HA/rGO-treated groups (> 75%) than that in the untreated group (62 ± 6%). HA/rGO also significantly improved the viability of the cells exposed to much higher concentrations of H_2_O_2_ (*i.e.*, 1 mM). While the untreated control group showed minimal viable cells (6 ± 3%), the HA/rGO-treated groups exhibited high cell viability in HA/rGO concentration dependent manner. Notably, the cells cultured with 100 μg/mL HA/rGO at 1 mM H_2_O_2_ presented the viability (73 ± 8%) similar to that cultured under normal condition (86 ± 1%), indicating a potent antioxidant capacity of HA/rGO. The potential cytotoxicity of HA/rGO was further evaluated using the lactate dehydrogenase (LDH) assay. In the ROS-free environment, HK2 cells incubated with HA/rGO with up to 100 μg/mL showed LDH levels comparable to those of the negative control, indicating a minimal cytotoxicity of HA/rGO (Figure S7A). HK2 cells cultured at 0.5 mM H_2_O_2_ in the presence of HA/rGO exhibited significantly lower LDH levels than untreated cells or cells treated with rGO (Figure S7B). Altogether, HA/rGO demonstrated antioxidant capacity to attenuate oxidative stress and protect cells under oxidative conditions.

### Paricalcitol loading, ROS-response release, and cellular uptake of HA/rGO

**Figure 3:**
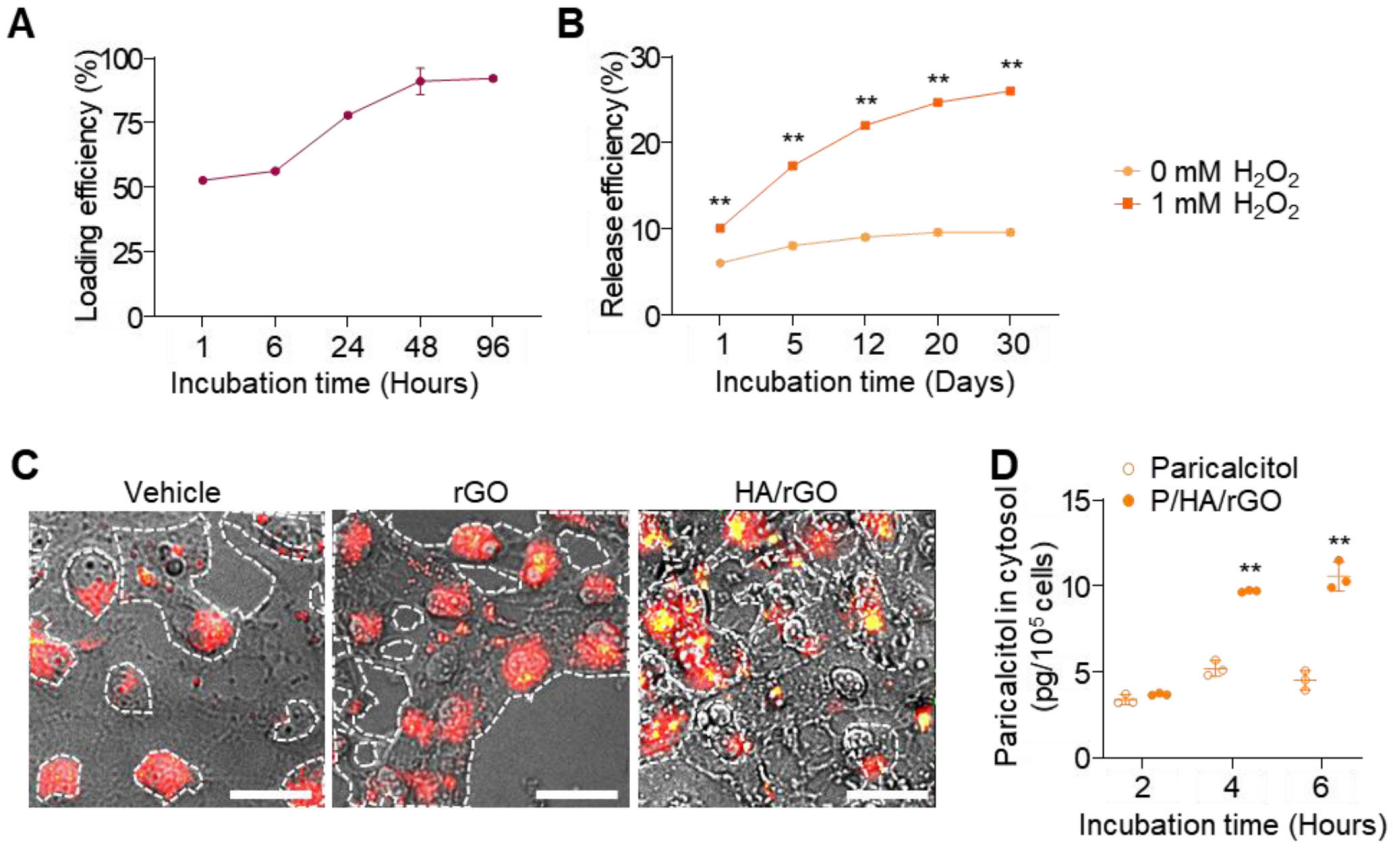
Drug loading, release, and intracellular delivery of P/HA/rGO. (A) Loading efficiency of paricalcitol loaded onto HA/rGO. (B) Paricalcitol release efficiency over 30 days under normal conditions or ROS conditions (1 mM H_2_O_2_). **P < 0.01 *vs.* 0 mM of H_2_O_2_. (C) Dark-field micrographs of HK2 cells treated with rGO or HA/rGO nanoparticles. Scale bars = 20 μm. (D) Intracellular paricalcitol levels in HK2 cells treated paricalcitol or P/HA/rGO. **P < 0.01 *vs.* paricalcitol.

Paricalcitol was efficiently loaded onto HA/rGO by incubation in paricalcitol solutions based on the hydrophobic interactions between paricalcitol and rGO. Paricalcitol loading steadily increased during the incubation and plateaued at 48 h, achieving a high loading efficiency of 93.0 ± 0.5% (Figure 3A). Accordingly, a 48-h incubation was selected for paricalcitol loading and preparation of P/HA/rGO in the following studies. The release behavior of paricalcitol from P/HA/rGO was examined under both normal (ROS-free) and oxidative (ROS-rich) conditions. Under normal condition, P/HA/rGO released a minimal amount of paricalcitol by day 20, resulting in a total release efficiency of 9.6 ± 0.4%, and no additional release was observed thereafter (Figure 3B). In contrast, under oxidative condition (at 1 mM H₂O₂), paricalcitol release from P/HA/rGO was significantly accelerated and prolonged for 30 days. For example, the release efficiency reached 26.0 ± 0.3% after 30 days incubation at 1 mM H₂O₂ (Figure 3B), which was 2.7-fold higher than that under ROS-free condition. These results demonstrate that oxidative microenvironment-induced release allows for selective and sustained drug delivery at injury sites where ROS levels are pathologically elevated.

Furthermore, alterations in CD44 expression in response to oxidative stress were examined by exposing HK2 cells to different H₂O₂ concentrations and immunostaining for CD44. CD44 expression in renal tubular epithelial cells increased as H₂O₂ concentrations increased (Figure S8), indicating that ROS upregulated CD44 expression and that HA-conjugated rGO could effectively target IR-injured renal tissues exhibiting high oxidative stress and CD44 expression. As the intracellular delivery of HA/rGO to renal tubular epithelial cells can substantially enhance the therapeutic efficacy of paricalcitol, the cellular uptake of HA/rGO nanoparticles was further examined using dark-field microscopy (Figure 3C). HK2 cells were pretreated with 1 mM H_2_O_2_ for 2 h and subsequently with nanoparticles (rGO or HA/rGO) for an additional 6 h. Untreated cells mostly showed red scattering, whereas cells treated with rGO cells displayed both red and yellow scattering within the cellular regions. In contrast, cells treated with HA/rGO demonstrated more pronounced and diverse yellow scattering signals in the red cellular regions, indicating efficient cellular uptake of HA/rGO. To further confirm intracellular drug delivery, paricalcitol levels were quantified in HK2 cells treated with either free paricalcitol or P/HA/rGO. The intracellular paricalcitol concentration in P/HA/rGO-treated cells was 2.3-fold higher than that in cells treated with free paricalcitol (Figure 3D).

### Biodistribution of HA/rGO after IR renal injury

**Figure 4:**
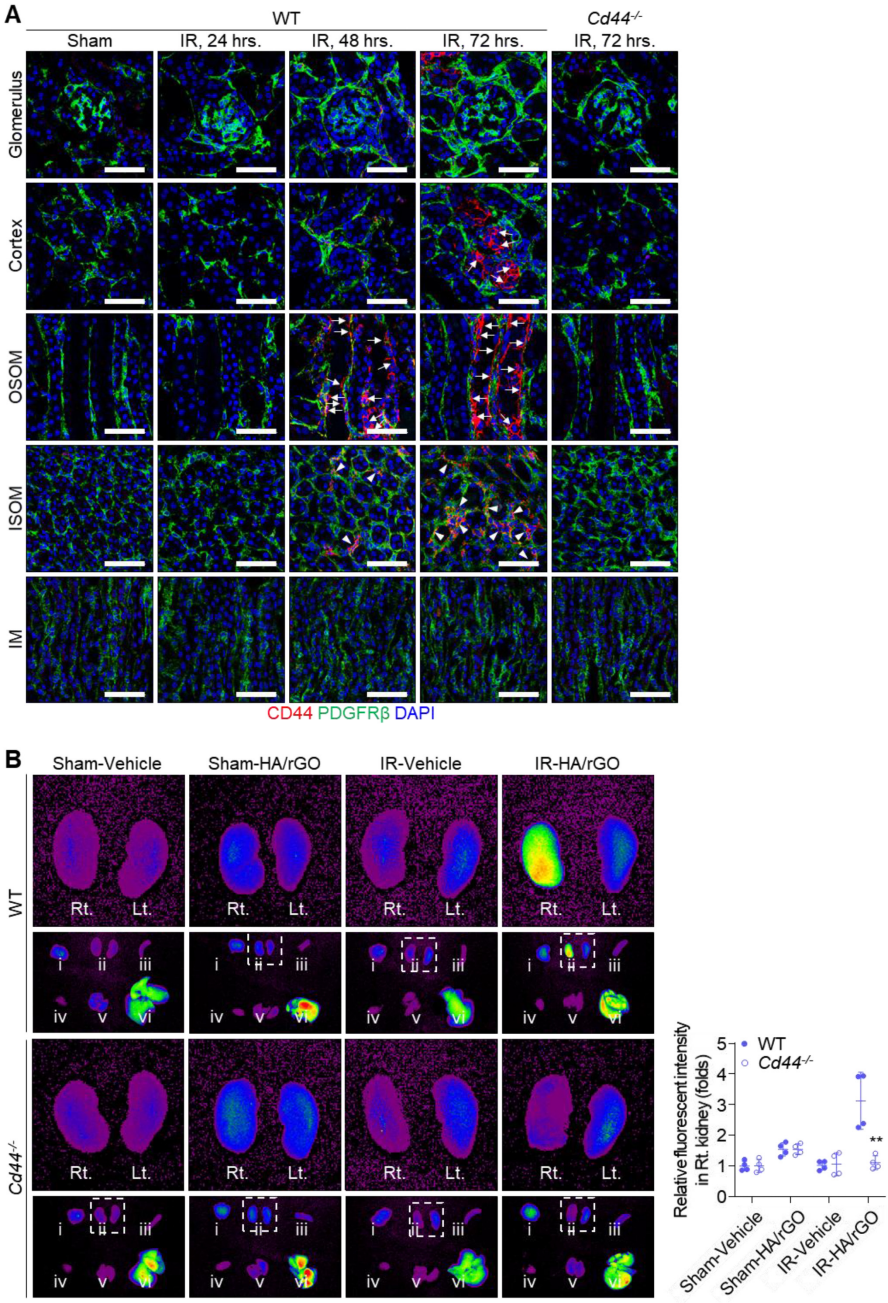
Biodistribution of HA/rGO after IR renal injury. (A) Representative immunostaining images for CD44 in the kidney after IR injury. The kidneys of wild type (WT) mice were harvested before (Sham) and at 24, 48, and 72 h after IR renal injury, and the kidneys of *Cd44^-/-^* mice were harvested at 72 h. Arrows and arrowheads indicate the high CD44 expression sites on basolateral side of tubules and in the interstitial compartment, respectively. IM, inner medulla; ISOM, inner stripe of outer medulla; OSOM, outer stripe of outer medulla. Scale bars = 20 μm. (B) The fluorescence images in various major organs, i) brain, ii) kidneys, iii) spleen, iv) liver, v) lung, and vi) heart, from WT and *Cd44^-/-^* mice at 48 h after IR renal injury and treatment with Cy5-labeled HA/rGO. The harvested organs are visualized in the lower panel. The right (Rt.) and left (Lt.) kidneys in the inset are magnified in the upper panel. For IR groups, Lt. and Rt. kidneys correspond to IR and normal contralateral kidneys, respectively. IR, ischemia/reperfusion. **P < 0.01 *vs.* WT.

Changes in CD44 expression in damaged kidney tissues were further examined *in vivo* by immunohistochemical staining of the IR renal injury mice (Figure 4A). No distinct CD44 expression was observed in the kidney sections until 24 h after IR renal injury. Surprisingly, 48 h after IR injury, a noticeable CD44 signal was detected on the basolateral side of the tubules in the outer stripe of the outer medulla (OSOM), and, to a lesser degree, in the interstitial compartment of the inner stripe of the outer medulla (ISOM). At 72 h after IR injury, more intense CD44 expression was observed in both the OSOM and ISOM of the kidney, and the basolateral side of the tubules in the cortex exhibited a strong CD44 signal. Even at this time point (72 h), CD44 expression in the glomerulus and inner medulla (IM) remained minimal. Importantly, no CD44 signal was observed in the kidneys of *Cd44*^-/-^ mice, even 72 h after IR renal injury.

To trace the biodistribution and kidney targeting capability of HA/rGO, various organs, including both kidneys, were harvested 48 h after IR renal injury and intravenous injection of Cy5-labeled HA/rGO. At 24 h following the injury, no distinct fluorescent signal was detected in the kidney (Figure S9A). At 48 h after injury, an intense fluorescence signal was detected in the IR-injured kidneys of wild-type (WT) mice (2.74 folds *vs.* vehicle-treated mice), while the fluorescence signal was minimally changed in the IR-injured kidneys of *Cd44^-/-^* mice, indicating that HA/rGO effectively targeted IR renal tissues via HA-CD44 interactions (Figure 4B). The fluorescent signals in contralateral kidneys and extrarenal organs (e.g., brain, spleen, heart, lung, and liver) also increased after IR renal injury (up to 2.00 folds *vs.* vehicle-treated mice), likely due to the decreased systemic clearance of the nanoparticles with kidney injury (Figure S9B). Altogether, the results demonstrated effective targeting of the IR/injured kidney using HA/rGO via CD44-mediated interactions.

### Therapeutic efficacy of P/HA/rGO in IR renal injury

**Figure 5:**
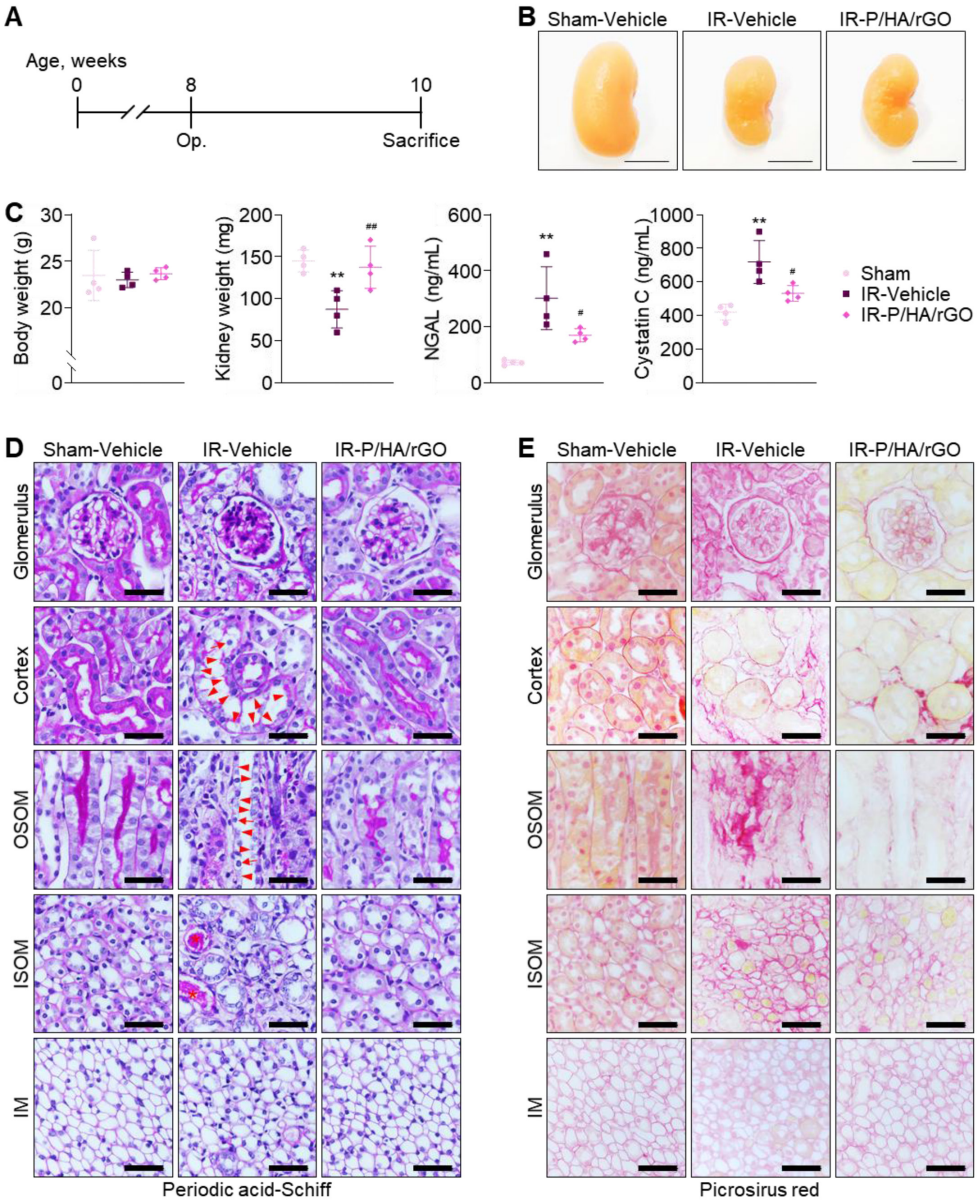
Functional and morphological evaluation of the therapeutic efficacy of P/HA/rGO in IR renal injury treatment. (A) Schematic of the experimental design for IR renal injury and treatment in mice. (B) Representative photographs of kidneys from the Sham-Vehicle, IR-Vehicle, and IR-P/HA/rGO groups at 2 weeks after post-injury or sham operation. (C) Body weights, kidney weights, and serum levels of NGAL and cystatin C (n = 4 mice/group). **P < 0.01 vs. Sham-Vehicle mice; ^#^*P* < 0.05, ^##^*P* < 0.01 *vs.* IR-Vehicle mice. (D) Periodic acid-Schiff and (E) Picrosirius red staining of the kidney sections. Scale bars = 20 μm. Arrows, arrowheads, and asterisks indicate mesangial expansion in glomeruli and exonucleation, loss of apical brush border, and intratubular cast formation in renal tubules, respectively. IM, inner medulla; IR, ischemia/reperfusion; ISOM, inner stripe of outer medulla; OSOM, outer stripe of outer medulla.

The therapeutic efficacy of P/HA/rGO for IR renal injury treatment was evaluated 2 weeks after injury (Figure 5A). The experimental groups included Sham-Vehicle, IR-Vehicle, and IR-P/HA/rGO. Visual inspection of the gross morphology of the kidneys indicated that IR injury led to kidney atrophy, which was slightly attenuated by the P/HA/rGO injection (Figure 5B). The body weights of the mice were not significantly different between the groups (Figure 5C). However, the kidney weights in IR-P/HA/rGO group (138 ± 25 mg) were significantly higher than those in the IR/Vehicle (88 ± 22 mg) group and similar to those in Sham-Vehicle group (145 ± 13 mg). Moreover, serum neutrophil gelatinase-associated lipocalin (NGAL) and cystatin C levels were significantly reduced by P/HA/rGO injection in the IR groups (IR-Vehicle and IR-P/HA/rGO) but were still higher than those in the Sham-Vehicle group (Figure 5C). For example, NGAL and cystatin C levels decreased from 302 ±112 ng/mL and 720 ± 128 ng/mL to 170 ± 23 ng/mL and 533 ± 47 ng/mL, respectively, by P/HA/rGO injection. Results indicate that P/HA/rGO facilitated the functional recovery of IR kidneys. Histological analyses with periodic acid-Schiff staining demonstrated that IR renal injury led to severe tissue damage, including mesangial expansion in glomeruli, exonucleation, loss of the apical brush border, and intratubular cast formation in renal tubules (Figure 5D). In addition, Picrosirius red staining revealed extensive interstitial fibrosis in the cortex, OSOM, and ISOM after IR injury (Figure 5E). Morphological alterations in the IM regions were not distinguishable, regardless of IR injury or P/HA/rGO injection. However, in other regnal regions, the injection of P/HA/rGO substantially reversed the morphological alterations caused by IR injury. Collectively, the injection of P/HA/rGO into mice demonstrated substantial protection of kidney function and morphology after IR renal injury.

**Figure 6:**
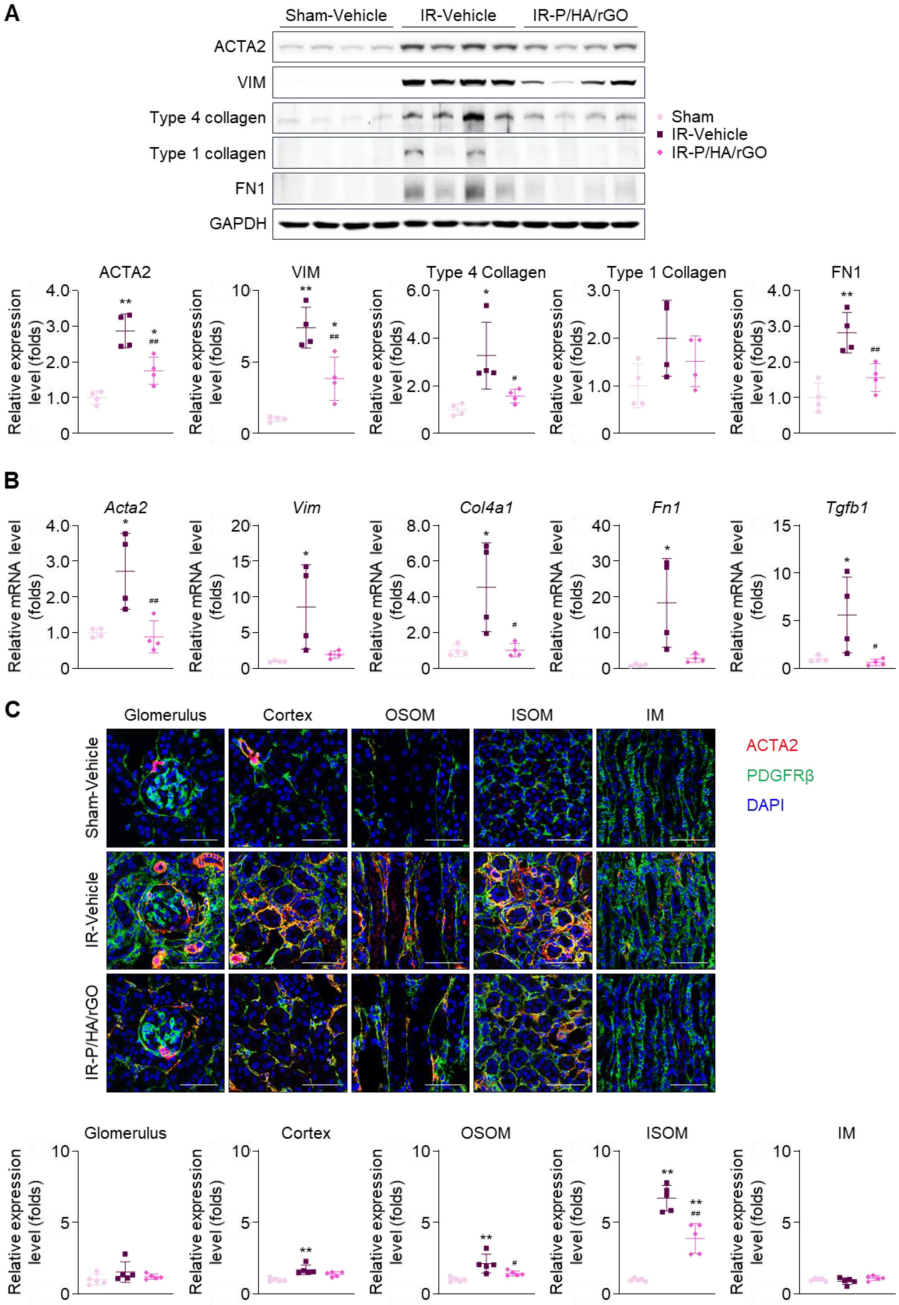
Effects of P/HA/rGO injection on renal fibrosis in IR injury. (A) Immunoblotting and quantification of fibrosis marker expression levels (ACTA2, vimentin (VIM), Type 4 collagen, Type 1 collagen, and fibronectin 1 (FN1) and GAPDH). Expression levels were demonstrated by normalizing the intensity of each marker to that of GAPDH in immunoblotting images. (B) RT-qPCR of the fibrosis marker genes in the Sham-Vehicle, IR-Vehicle, and IR-P/HA/rGO groups (n = 4 mice/group). GAPDH was applied as the internal control for both immunoblotting and RT-qPCR. (C) Immunohistochemical staining for ACTA2, PDGFRβ, and DAPI (nucleus) in the kidneys (n = 5 mice/group). Scale bars = 20 μm. **P < 0.01 *vs.* Sham-Vehicle mice; ^#^*P* < 0.05, ^##^*P* < 0.01 vs. IR-Vehicle mice. IM, inner medulla; IR, ischemia/reperfusion; ISOM, inner stripe of outer medulla; OSOM, outer stripe of outer medulla.

The anti-fibrotic effects of P/HA/rGO on kidney tissues were further examined by immunoblotting, RT-qPCR, and immunohistochemistry (Figure 6). Immunoblotting of kidney tissue lysates revealed significant upregulation of the expression of fibrosis markers (*i.e.*, ACTA2, vimentin, type IV collagen, and fibronectin) following IR renal injury (Figure 6A). RT-qPCR demonstrated that the corresponding genes, *Acta2*, *Vim*, *Col4a1*, *Fn1*, and *Tgbf1* were significantly upregulated in the IR-Vehicle group (Figure 6B). Notably, *Tgbf1* encodes TGFβ, a key profibrotic cytokine. Overall, P/HA/rGO injection significantly attenuated the expression of these fibrotic markers to levels comparable to those in the Sham-Vehicle group. The gene expression of *Acta2*, *Vim*, *Col4a1*, *Fn1*, and *Tgbf1* in the IR-P/HA/rGO groups decreased by 67.4%, 77.3%, 77.7%, 84.6%, and 88.7%, respectively, compared with those in the IR-Vehicle group (Figure 6A-B). Immunostaining for PDGFRβ revealed that its signals were not significantly different among the groups (Figure 6C). The expression of ACTA2 was significantly increased in the kidneys of the IR injury groups, and ACTA2 signals were restricted to the glomerular parietal epithelium and interstitial compartments of the cortex, OSOM, and ISOM, sparing the glomerular mesangium and IM regions. P/HA/rGO significantly suppressed the upregulation of ACTA2 in the kidneys. In IR-injured mice treated with P/HA/rGO, the relative expression of ACTA2 in the cortex, OSOM, and ISOM decreased from 1.7 ± 0.3, 2.1 ± 0.7, and 6.7 ± 0.9-fold to 1.4 ± 0.2, 1.4 ± 0.2, and 3.9 ± 1.0-fold, respectively.

**Figure 7:**
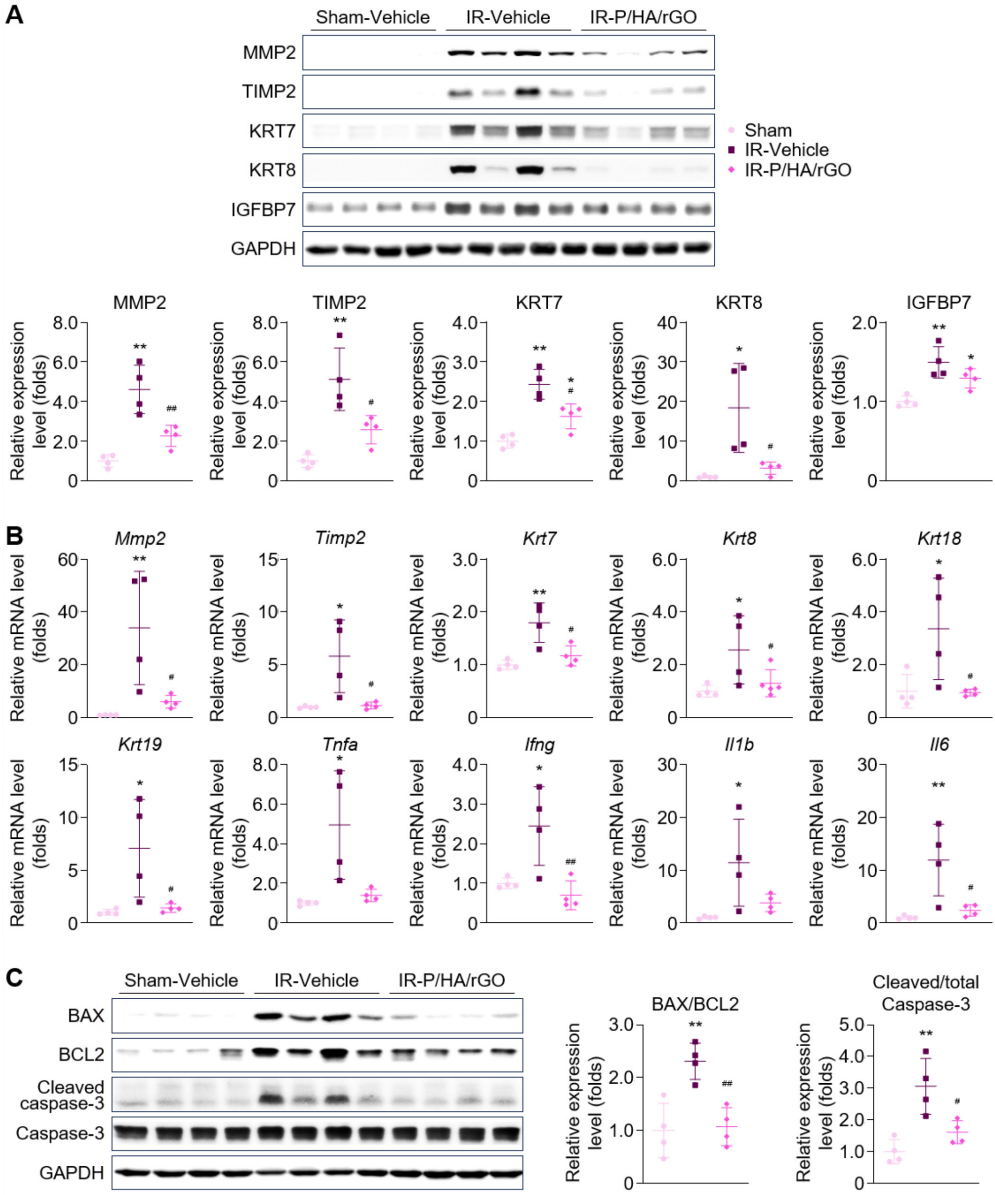
Suppressive effect of P/HA/rGO on tubular injury, tissue inflammation, and apoptosis after IR renal injury. (A) Immunoblotting and (B) RT-qPCR analyses of the kidney lysate from Sham-Vehicle, IR-Vehicle, and IR-P/HA/rGO groups for the expression of the tubular injury and tissue inflammation markers (*n* = 4 mice/group). Expression levels were normalized to GAPDH for both immunoblotting and RT-qPCR analyses. (C) Immunoblotting analysis of apoptotic markers in kidney lysates from Sham-Vehicle, IR-Vehicle, and IR-P/HA/rGO groups (*n* = 4 mice/group). GAPDH was applied as the internal control for all experiments. *P < 0.05, **P < 0.01 *vs.* Sham-Vehicle mice; ^#^*P* < 0.05, ^##^*P* < 0.01 *vs.* IR-Vehicle mice.

Furthermore, the direct inhibitory effect of P/HA/rGO on fibroblast activation was evaluated *in vitro* by treating TGFβ-stimulated NRK49F cells with P/HA/rGO (Figure S10). TGFβ stimulation significantly induced the phosphorylation of SMAD2/3 and increased the expressions of fibrosis markers (*i.e.,* fibronectin, type I collagen, and ACTA2). Cotreatment with P/HA/rGO effectively attenuated SMAD2/3 phosphorylation (by 83.8%) and the expression of fibrosis markers (by 81.6% for fibronectin and 56.1% for ACTA2). The effects of P/HA/rGO on the pathological processes leading to fibrosis were further examined *in vivo* by analyzing renal tubular injury, tissue inflammation, and apoptosis, which are hallmarks of the early phase of the AKI-to-CKD transition. Immunoblotting analysis demonstrated a significant upregulation of renal tubular injury markers in the IR-Vehicle group compared with that in the Sham-Vehicle group (Figure 7A). RT-qPCR analyses also demonstrated significant upregulation of genes associated with renal tubular injury (*Mmp2*, *Timp2*, *Krt7*, *Krt8*, *Krt18*, and *Krt19*) and pro-inflammatory cytokines (*Tnfa*, *Ifng*, *Il1b*, and *Il6*) (Figure 7B).

Treatment with P/HA/rGO significantly mitigated the abnormal molecular expression associated with renal tubular injury and tissue inflammation caused by IR injury, restoring levels close to those observed in the Sham group (Figure 7A-B). For example, the signal intensities of MMP2, TIMP2, KRT7, KRT8, and IGFBP7 in the IR-P/HA/rGO group were reduced by 50.9%, 49.6%, 33.3%, 82.7%, and 13.4%, respectively, compared with those in the IR-Vehicle group. Moreover, immunoblotting for apoptotic markers (BAX, BCL2, cleaved caspase, and total caspase-3) revealed significant increases in the BAX-to-BCL2 and cleaved-to-total caspase-3 ratios in the IR-Vehicle group. Apoptosis induced by renal IR injury was effectively suppressed by P/HA/rGO treatment to a level comparable to that in the Sham group (Figure 7C), as the BAX-to-BCL2 and the cleaved-to-total caspase-3 ratios decreased by 53.5% and 47.3%, respectively, in the IR-P/HA/rGO group compared with those in the IR-Vehicle group. Morphological analyses of the extrarenal organs showed no substantial pathological changes in the IR renal injury groups, regardless of co-treatment with P/HA/rGO (Figure S11). These results collectively demonstrate that P/HA/rGO effectively suppresses key profibrotic processes, including fibroblast activation, tubular injury, inflammation, and apoptosis, and thereby mitigates early pathological events.

## Discussion

The progression of AKI to CKD remains a major clinical concern due to the absence of effective therapeutic strategies that can address complex and multifactorial pathogenesis of AKI-to-CDK transition 28. In this study, we developed a multifunctional therapeutic nanoplatform capable of targeting injured kidneys, scavenging ROS, and releasing anti-fibrotic agents in response to oxidative stimuli for preventing the AKI-to-CKD transition and restoring renal functions (Scheme 1). P/HA/rGO demonstrated multifunctional therapeutic effects both *in vitro* and *in vivo*. We synthesized uniform, well-dispersed, and stable HA-conjugated rGO nanoparticles via *in situ* reduction of GO in an HA solution using ascorbic acid and high-power sonication (Figure 1), which enabled covalent functionalization of GO with HA 29-31. Our synthesis method, permitting simultaneous GO reduction and HA conjugation, was developed by adapting previous reports. For instance, Cruz-Benitez et al. reported that high-power sonication of GO can induce covalent functionalization with carbohydrates, such as fructose and starch 29. Moreover, mild reduction of GO to rGO by ascorbic acid induces partial restoration of sp^2^ carbon structures, as evidenced by increased I_D_/I_G_ ratios in Raman spectroscopy (Figure 1E) 26. These structural changes increase antioxidant properties (Figure 1G-H) and hydrophobicity of the rGO. Enhanced antioxidant activities of rGO have been widely reported in literature 12. In particular, enhanced hydrophilicity increased the affinity to hydrophobic drugs (e.g., paricalcitol) (Figure 3A). Furthermore, the quantification of residual ascorbic acid in HA/rGO after synthesis and purification indicated minimal residual levels. This minimal residue likely resulted from extensive washing procedures during purification, effectively removing hydrophilic ascorbic acid. Hence, the enhanced ROS-scavenging activities of rGO/HA appeared to be primarily attributed to the rGO component produced by ascorbic acid-medicated reduction of GO.

Physicochemical characterization indicated that HA conjugation improved the colloidal stability and biocompatibility of rGO, while providing kidney-targeting ability via CD44 interactions. HA/rGO nanoparticles effectively scavenged ROS and protected renal tubular epithelial cells from oxidative stress. Efficient cellular uptake of P/HA/rGO was also observed under oxidative stress conditions. Since the vitamin D receptor, the target of paricalcitol, is localized in the cytosol 32, enhanced cellular uptake of P/HA/rGO is beneficial for increasing intracellular paricalcitol level and promoting its biological effects.

Paricalcitol was selected for its well-established anti-fibrotic effects via vitamin D receptor-mediated inhibition of TGF-β/SMAD signaling 17. Paricalcitol can be clinically used to manage secondary hyperparathyroidism in kidney diseases due to its anti-fibrotic and anti-inflammatory effects. However, its clinical application is limited by poor solubility, systemic side effects, and pharmacokinetic challenges. In our *in vitro* study, paricalcitol was efficiently loaded paricalcitol onto HA/rGO nanoparticles (93.0 ± 0.5% loading efficiency) through strong hydrophobic interactions between paricalcitol and rGO (Figure 3A). Minimal drug release (9.6 ± 0.4% over 30 days) under non-oxidative conditions (Figure 3B) further supports the strong interactions between rGO and paricalcitol. P/HA/rGO exhibited an ROS-inducible paricalcitol release behavior (Figure 3), likely attributed to ROS-induced oxidation of rGO. This oxidative disruption of the sp^2^ hybridized structure of rGO decreases its hydrophobicity 33, 34, reducing its affinity for hydrophobic paricalcitol and facilitating paricalcitol release. Although we have not directly evaluated paricalcitol release *in vivo*, we expect ROS-triggered paricalcitol release from P/HA/rGO in IR kidneys, in which ROS levels are elevated and P/HA/rGO accumulates through HA-CD44-mediated targeting.

Biodistribution studies demonstrated that HA-CD44 interactions were essential for the kidney-targeting ability of HA/rGO (Figure 4). HA/rGO selectively accumulated in IR-injured kidneys while exhibiting minimal accumulation in other organs (Figure S9), highlighting the specificity of HA-CD44 targeting. Interestingly, we observed that the kidney-specific accumulation of HA/rGO occurred between 24 to 48 h after IR injury (specifically 18 h to 42 h after intravenous injection of HA/rGO) (Figure 4B-S9A). This observation implies that a substantial portion of the injected HA/rGO particles remained in systemic circulation until this time point. In other words, this strongly suggests that the injected HA/rGO particles have successfully escaped potential elimination pathways, including the glomerular filtration, at least until 48 h after IR injury. Therefore, glomerular filtration appears unlikely as a primary route for the kidney-specific delivery of HA/rGO. Instead, we postulate that peritubular capillaries may serve as the major pathway of the kidney-specific delivery of HA/rGO. Although fenestrations exist along the peritubular capillaries under physiological conditions, their size is slightly smaller than those of glomeruli 35,36. However, it is well established that capillary leakage significantly increases after tissue injury 37, indicating that the normal cut-off size of capillary fenestrations may be no more applicable in injured tissues. Moreover, the utilization of the peritubular capillary fenestrations for the kidney-specific delivery of HA/rGO offers additional advantages, because CD44 is specifically expressed along the basolateral surfaces of tubular epithelial cells. If HA/rGO had penetrated the glomerular filtration barrier, the apical expression of CD44 on tubular epithelial cells would have been advantageous; however, this scenario is not supported by our results. In contrast, CD44 expression was localized on the basolateral surfaces interfacing interstitial spaces, thus maximizing the possibility that HA/rGO particles “leaked” from peritubular capillaries could effectively bind to CD44. This HA/rGO nanocarrier allowed for selective delivery of paricalcitol to IR-injured kidneys while mitigating its extra-renal effects (*e.g.*, hypercalcemia) and maximizing drug concentration at fibrotic sites. The HA-CD44-mediated targeting mechanism offers several merits for the clinical application of HA/rGO, including enhanced drug retention in injured kidneys and reduced off-target effects 38, 39. In addition, the prolonged circulation time of HA/rGO permits a flexible therapeutic window (48-72 h after IR injury) for AKI treatment.

The therapeutic efficacy of P/HA/rGO in halting AKI-to-CKD progression was confirmed by histological and molecular analyses. Treatment with P/HA/rGO effectively reduced SMAD2/3 phosphorylation (Figure S10) and suppressed the expression of fibrosis-associated proteins, including fibronectin, type I collagen, and ACTA2 (Figure 6), suggesting the ability of HA/rGO to directly modulate fibrotic signaling cascades. *In vitro* studies indicated that P/HA/rGO inhibited TGFβ-induced fibroblast activation, reducing extracellular matrix deposition and fibrotic remodeling (Figure S10). Immunohistochemical analysis further confirmed the significant suppression of fibrotic markers in P/HA/rGO-treated kidneys compared with that of the untreated IR-injured controls (Figure 6). Note that we used *Cd44^-/-^* mice to validate that the accumulation of HA/rGO in the kidneys was CD44-mediated. However, the effects of intravenous injection of P/HA/rGO on therapeutic efficacy in *Cd44^-/-^* mice were not assessed. As the knockout of CD44 is known to be protective against various types of kidney injury including IR injury 40, 41, we presumed that intravenously injected P/HA/rGO may result in comparable therapeutic efficacy in *Cd44^-/-^* mice, even without specific kidney targeting. To clarify data interpretation, this experimental set was excluded from the present study.

While other antioxidant or anti-fibrotic nanoplatforms have been reported, P/HA/rGO offers unique benefits by integrating targeted delivery with ROS-responsive drug release. The HA moiety facilitates accumulation in injured kidneys, while ROS-triggered paricalcitol release enables spatiotemporally controlled therapy. The combined suppression of oxidative damage, inflammation, and fibrosis is critical for halting the AKI-to-CKD transition. Importantly, our HA/rGO nanomaterial platform supports the loading and ROS-responsive release of other hydrophobic drugs possessing physicochemical properties (e.g., hydrophobicity) similar to those of paricalcitol 42, 43, expanding its potential for broader therapeutic applications. This study presents a promising strategy for the treatment of IR-induced AKI by leveraging a multifunctional, kidney-targeted, and ROS-responsive nanoplatform. Although the present study demonstrated the therapeutic potential of HA/rGO for IR renal injury, additional research is necessary to comprehensively assess its long-term safety and biocompatibility. In particular, investigations into chronic toxicity, immune responses, and biodistribution over extended periods are critical for clinical translation.

## Conclusion

In this study, we developed and evaluated a multifunctional nanoplatform (P/HA/rGO) to target IR-induced AKI and prevent the AKI-to-CKD transition. This novel nanoplatform, composed of rGO, HA, and paricalcitol, was produced by *in situ* mild chemical reduction and sonication. HA offers kidney-specific targeting via CD44 interactions, whereas rGO provided robust antioxidant activity and enabled ROS-responsive drug release. The results demonstrated that P/HA/rGO effectively scavenged ROS, reduced tubular cell apoptosis, and significantly mitigated inflammation *in vitro* and *in vivo*. ROS-triggered release of paricalcitol significantly inhibited fibroblast activation and renal fibrosis in IR-injured kidneys. Biodistribution studies confirmed that the selective accumulation of P/HA/rGO in injured renal tissues was mediated by HA-CD44 binding. Histological and molecular analyses revealed that P/HA/rGO treatment preserved renal morphology, recovered renal function, suppressed profibrotic marker expression, and restored tissue homeostasis. Our findings highlight P/HA/rGO as a promising therapeutic strategy for the treatment of AKI-to-CKD transition following IR injury, leveraging targeted delivery and oxidative stress-responsive drug release. This platform opens new avenues for nanomedicine in the treatment of kidney diseases.

## Supplementary Material

Supplementary figures and tables.

## Abbreviations

AKI: Acute kidney injury
CKD: chronic kidney disease
ROS: reactive oxygen species
HA: hyaluronic acid
rGO: reduced graphene oxide
HA/rGO: HA conjugated rGO nanoparticle
IR: ischemia/reperfusion
ESKD: end-stage kidney disease
P/HA/rGO: paricalcitol loaded HA conjugated rGO nanoparticle
TEM: transmission electron microscopy
FTIR: fourier transform infrared
TGA: thermogravimetric analysis
PDI: polydispersity index
DPPH: 2,2-diphenyl-1-picrylhydrazyl
3-ethylbenzothiazoline-6-sulfonic acid): ABTS, 2,2′-azino-bis
LDH: lactate dehydrogenase
OSOM: outer stripe of the outer medulla
ISOM: inner stripe of the outer medulla
IM: inner medulla
WT: wild-type
NGAL: neutrophil gelatinase-associated lipocalin
