# Supplementary material for: ROS-responsive graphene-hyaluronic acid nanomedicine for targeted therapy in renal ischemia/reperfusion injury
Source: Theranostics. 2026 Jan 1;16(2):618–36. doi: 10.7150/thno.120560 (PMC12674889; doi:10.7150/thno.120560)
Supplement: Supplementary file 1 — Supplementary figures and tables. [file thnov16p0618s1.pdf]

Supplementary Information

ROS-Responsive Graphene–Hyaluronic Acid Nanomedicine for Targeted Therapy in Renal Ischemia/Reperfusion Injury

Seungjun Lee[1,#], Sang Heon Suh[2,#], Seong Kwon Ma[2], Junghyun Kim [1], Sehyeon Park [1], Soo Wan Kim[2,*] and Jae Young Lee [1,*]

[1]School of Materials Science and Engineering, Gwangju Institute of Science and Technology (GIST), Gwangju 61005, Republic of Korea

[2]Department of Internal Medicine, Chonnam National University Medical School, Gwangju, 61469, Republic of Korea

[#] These authors contributed equally to this work.

* Corresponding authors: e-mail to jaeyounglee@gist.ac.kr (JYL), skimw@chonnam.ac.kr (SWK)

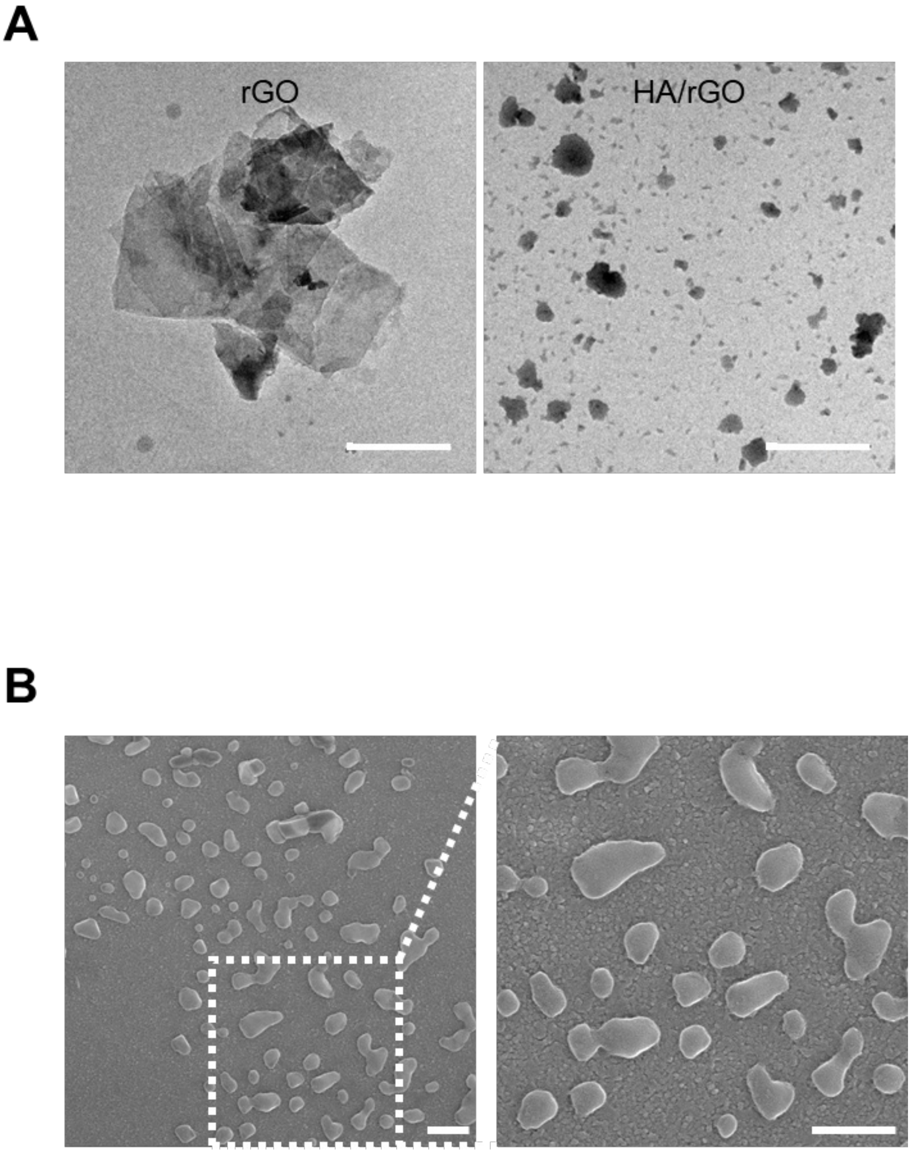

Figure S1. Microscopy images of nanoparticles (A) Transmission electron microscopy images of rGO and HA/rGO and (B) scanning electron microscopy image of P/HA/rGO. Scale bars = 0.5 μm. HA, hyaluronic acid; rGO, reduced graphene oxide; P, Paricalcitol

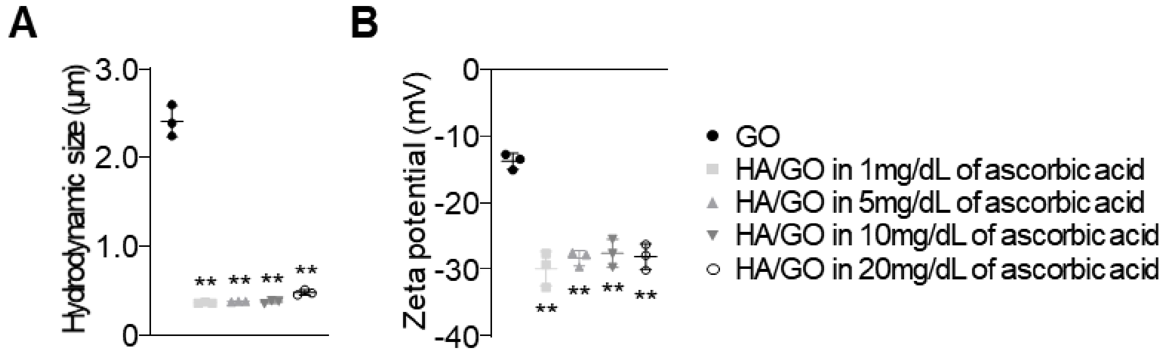

Figure S2. Effects of ascorbic acid concentrations during HA/rGO synthesis on (A) hydrodynamic sizes and (B) zeta potential measurement. ***P* < 0.01 *vs.* GO by one-way ANOVA with Tukey's multiple comparison tests. HA, hyaluronic acid; GO, graphene oxide; rGO, reduced graphene oxide

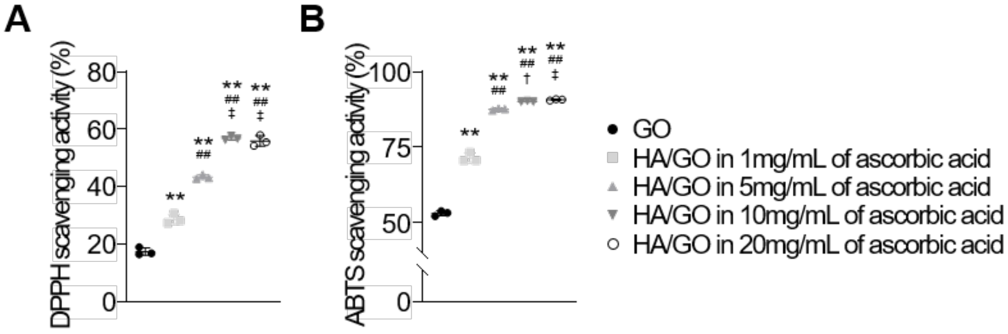

Figure S3. Radical scavenging activities of GO and HA/rGO synthesized with various ascorbic acid concentrations (1, 5, 10, and 20 mg/mL). (A) DPPH radical scavenging activity and (B) ABTS radical scavenging activity. $^{**}P < 0.01$ *vs.* GO; $^{\#\#}P < 0.01$ *vs.* HA/GO in 1 mg/dL of ascorbic acid; $^{\dagger}P < 0.05$, $^{\ddagger}P < 0.01$ *vs.* HA/GO in 5 mg/dL of ascorbic acid by one-way ANOVA with Tukey's multiple comparison tests. HA, hyaluronic acid; GO, graphene oxide; rGO, reduced graphene oxide

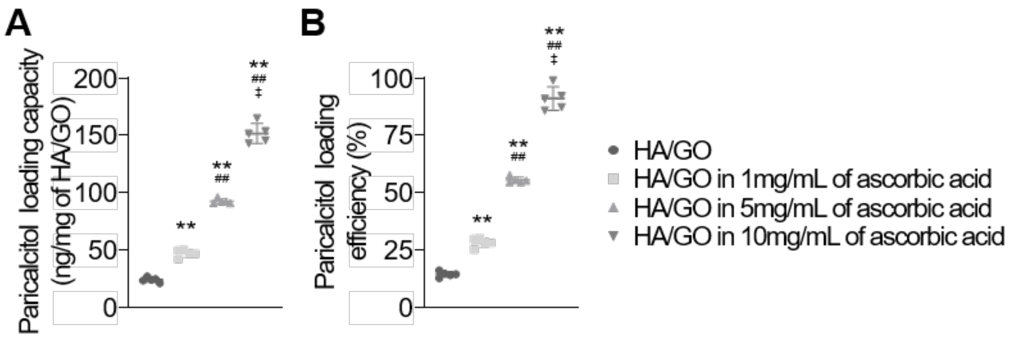

Figure S4. Paricalcitol levels loaded onto HA/GO and HA/rGO synthesized with various ascorbic acid concentrations (1, 5, and 10 mg/mL). (A) Paricalcitol loading capacity and (B) paricalcitol loading efficiency. $^{**}P < 0.01$ *vs.* HA/GO; $^{\#\#}P < 0.01$ *vs.* HA/GO in 1 mg/mL of ascorbic acid; $^{‡}P < 0.01$ *vs.* HA/GO in 5 mg/mL of ascorbic acid by one-way ANOVA with Tukey's multiple comparison tests. HA, hyaluronic acid; GO, graphene oxide; rGO, reduced graphene oxide

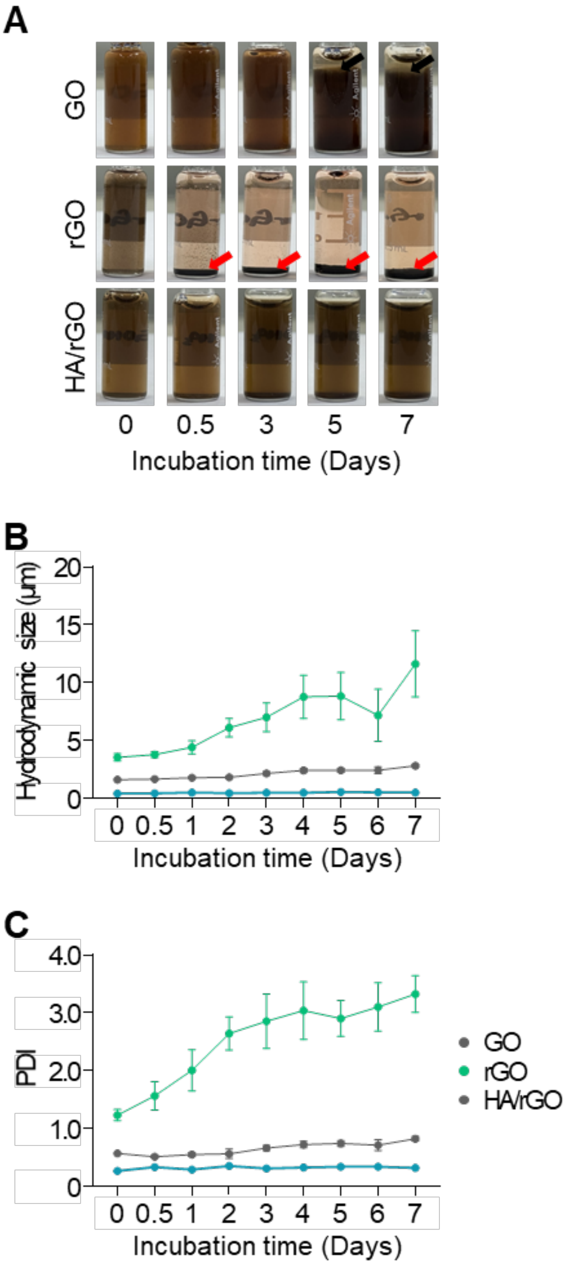

Figure S5. Colloidal stability of GO, rGO, and HA/rGO in FBS-containing medium. (A) Photographs demonstrating the stability of GO, rGO, and HA/rGO in 10% FBS-containing medium at 37°C for up to 7 days. Red arrows indicate sediments of nanoparticles. (B) Hydrodynamic size and (C) polydispersity index (PDI) of the nanoparticle solutions during the incubation periods. FBS, fetal bovine serum; HA, hyaluronic acid; GO, graphene oxide; rGO, reduced graphene oxide

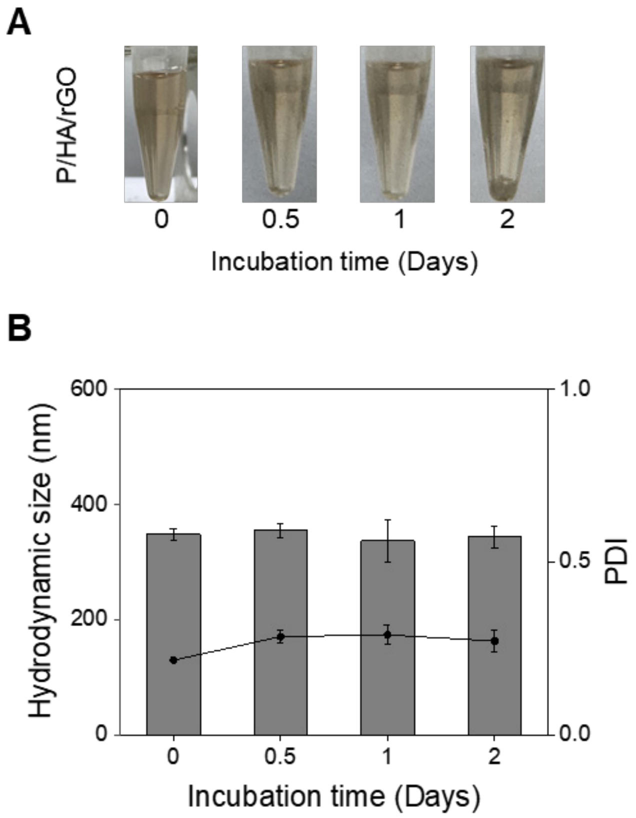

Figure S6. Colloidal stability of P/HA/rGO under physiological condition. (A) Photographs demonstrating the stability P/HA/rGO in 50% mouse blood serum at 37°C for up to 2 days in the 200-rpm shaking incubator. (B) Hydrodynamic size and polydispersity index (PDI) of the nanoparticle solutions during the incubation periods. FBS, fetal bovine serum; HA, hyaluronic acid; rGO, reduced graphene oxide; P, Paricalcitol drug

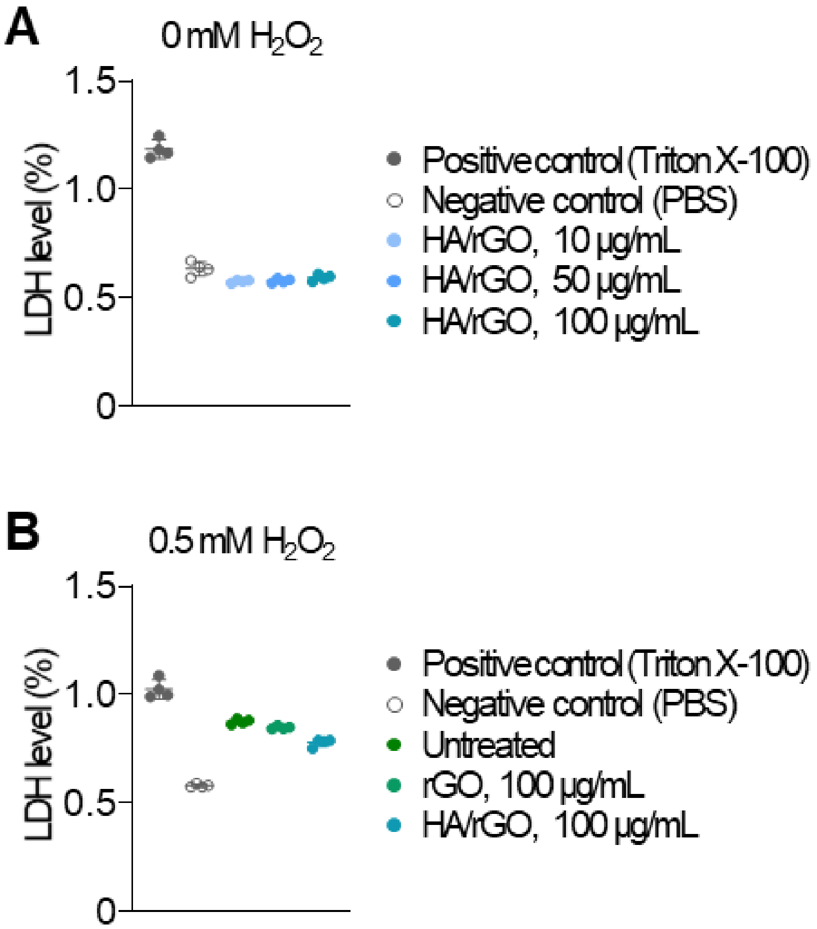

Figure S7. Cytotoxicity tests of HA/rGO using HK2 cells. (A) LDH levels of HK2 cells exposed to various HA/rGO concentrations (10, 50, and 100 μg/mL) under normal conditions. (B) LDH levels of HK2 cells exposed to 100 μg/mL of rGO or HA/rGO under ROS conditions (0.5 mM H_2O_2). Media from HK2 cells treated with Triton X-100 was used as the positive control, and media from untreated HK2 cells was used as the negative control. *$P < 0.05$ *vs.* negative control by one-way ANOVA with Tukey's multiple comparison tests. **$P < 0.01$ *vs.* negative control; $^{\#\#}P < 0.01$ *vs.* untreated group; $^{\ddagger}P < 0.01$ *vs.* 100 μg/mL of rGO. HA, hyaluronic acid; LDH, lactate dehydrogenase; rGO, reduced graphene oxide

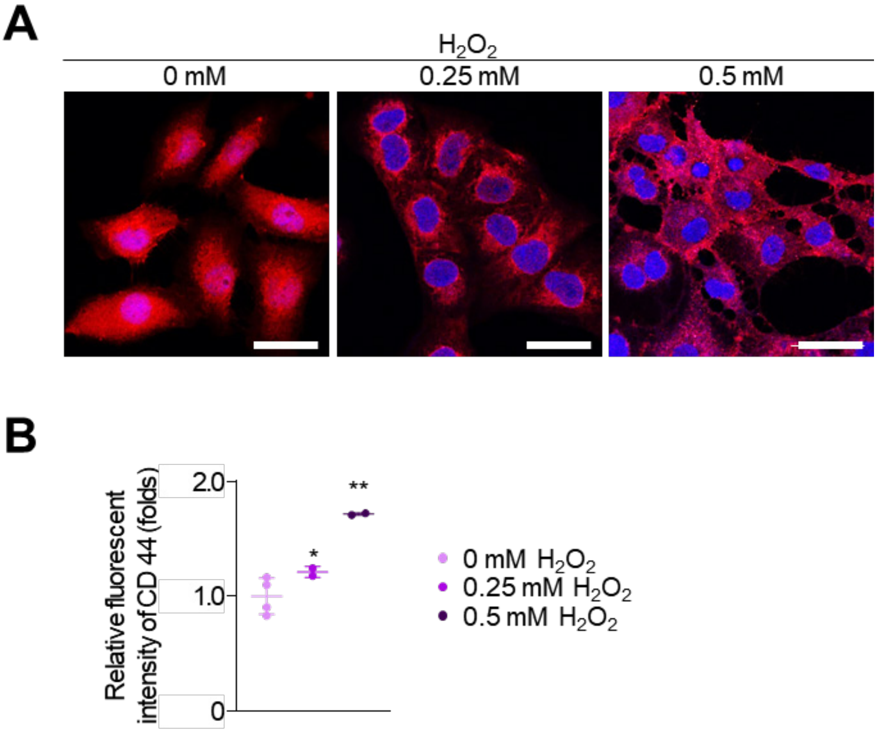

Figure S8. *In vitro* expression of CD44 in HK2 cells. (A) Immunostaining for CD44 of HK2 cells cultured with various H_2O_2 concentrations (0, 0.25, and 0.5 mM). Red and blue colors represent CD44 and nuclei, respectively. Scale bars = 40 μm. (B) Relative fluorescence intensity of CD44. **$P < 0.01$ *vs.* the cells cultured at 0 mM H_2O_2; $^{\#}P < 0.05$ *vs.* the cells cultured at 0.25 mM H_2O_2.

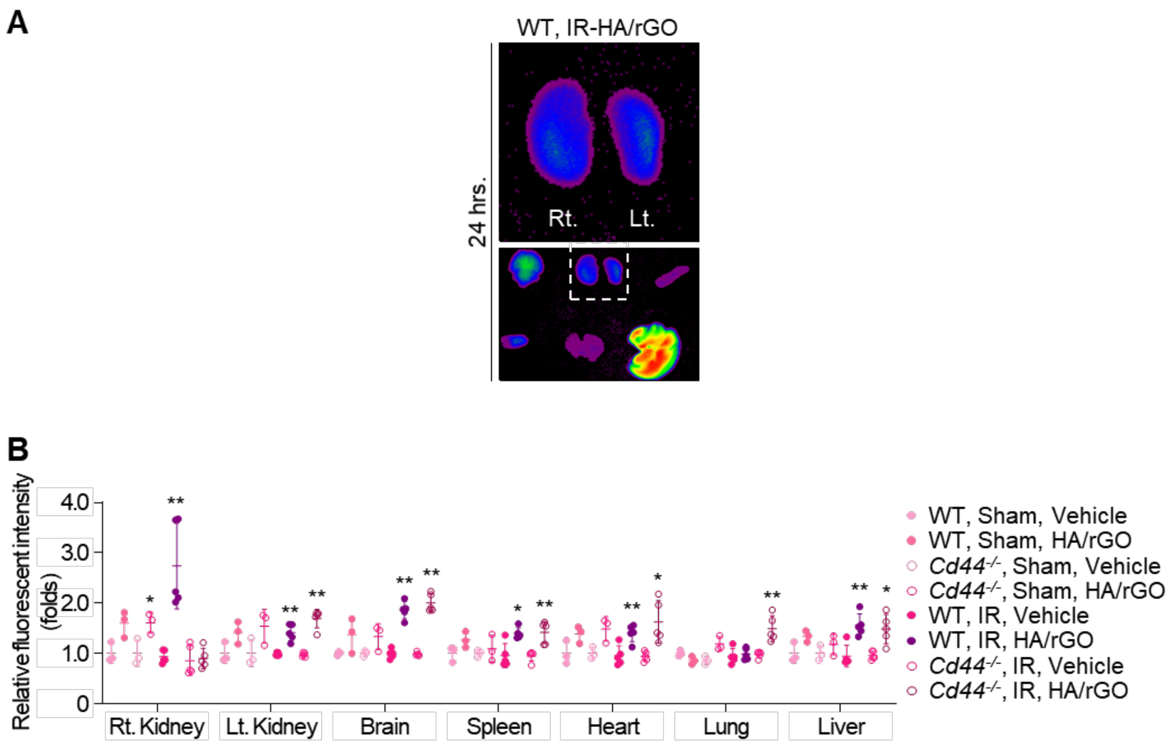

Figure S9. Biodistribution for HA/rGO in various organs. (A) The fluorescence images of various organs in wild type mice 24 h after IR renal injury and Cy5-labeled HA/rGO injection. The harvests organs (brain, kidneys, spleen, liver, lung, and heart) are visualized in the left panel (clockwise from upper left). The right (Rt.) and left (Lt.) kidneys in the inset are magnified in the right panel. (B) Quantification of Cy5-labeled HA/rGO signals from the various organs. The fluorescence intensities were normalized to the mean value of the vehicle-treated mice. *$P < 0.05$; **$P < 0.01$ *vs.* vehicle-treated mice by Student's t-test. HA, hyaluronic acid; IR, ischemia/reperfusion; rGO, reduced graphene oxide

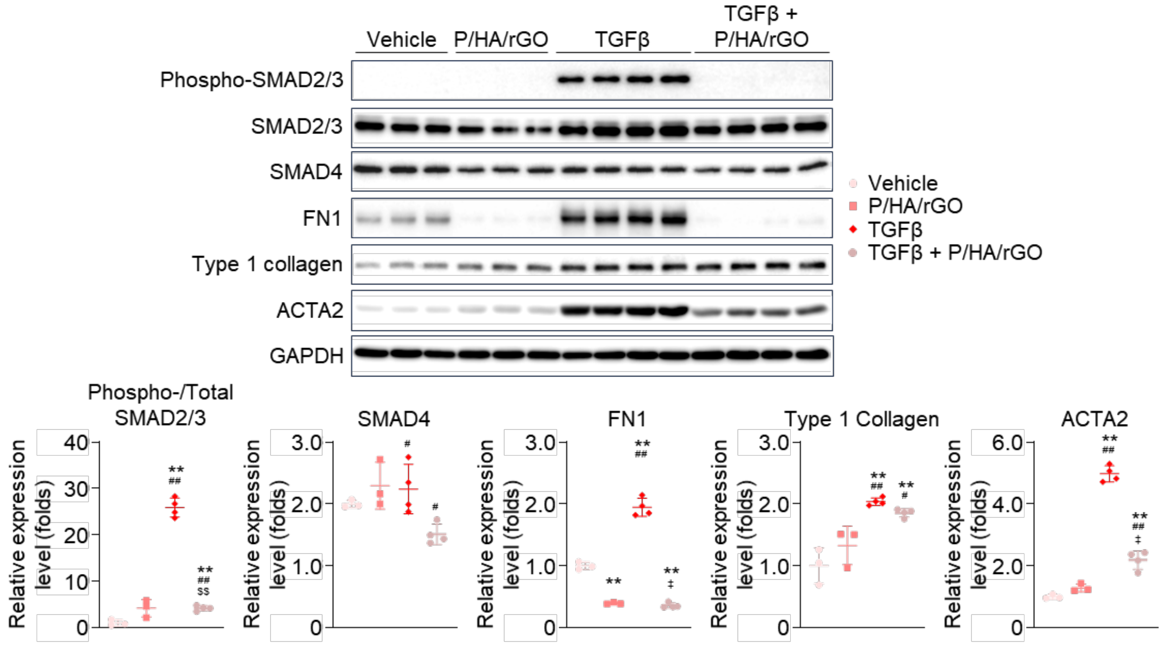

Figure S10. Suppression of TGFβ-induced fibroblast activation by P/HA/rGO. NRK49F cells (a rat interstitial fibroblast cell line) were treatment with TGF-β1 (5 ng/ml) for 24 hours with (TGFβ + P/HA/rGO group) or without (TGFβ group) the pretreatment of P/GO/HA (50 μg/mL) 1 hour prior to TGF-β1 treatment. Negative controls included cells treated only by PBS (Vehicle group) and by P/HA/rGO without cotreatment of with TGF-β1 (P/HA/rGO group) (n = 3~4 dishes/group). $^{**}P < 0.01$ *vs.* Vehicle group; $^{\#}P < 0.05$, $^{\#\#}P < 0.01$ *vs.* P/HA/rGO group; $^{\ddagger}P < 0.01$ *vs.* TGFβ group by one-way ANOVA with Tukey's multiple comparison tests.

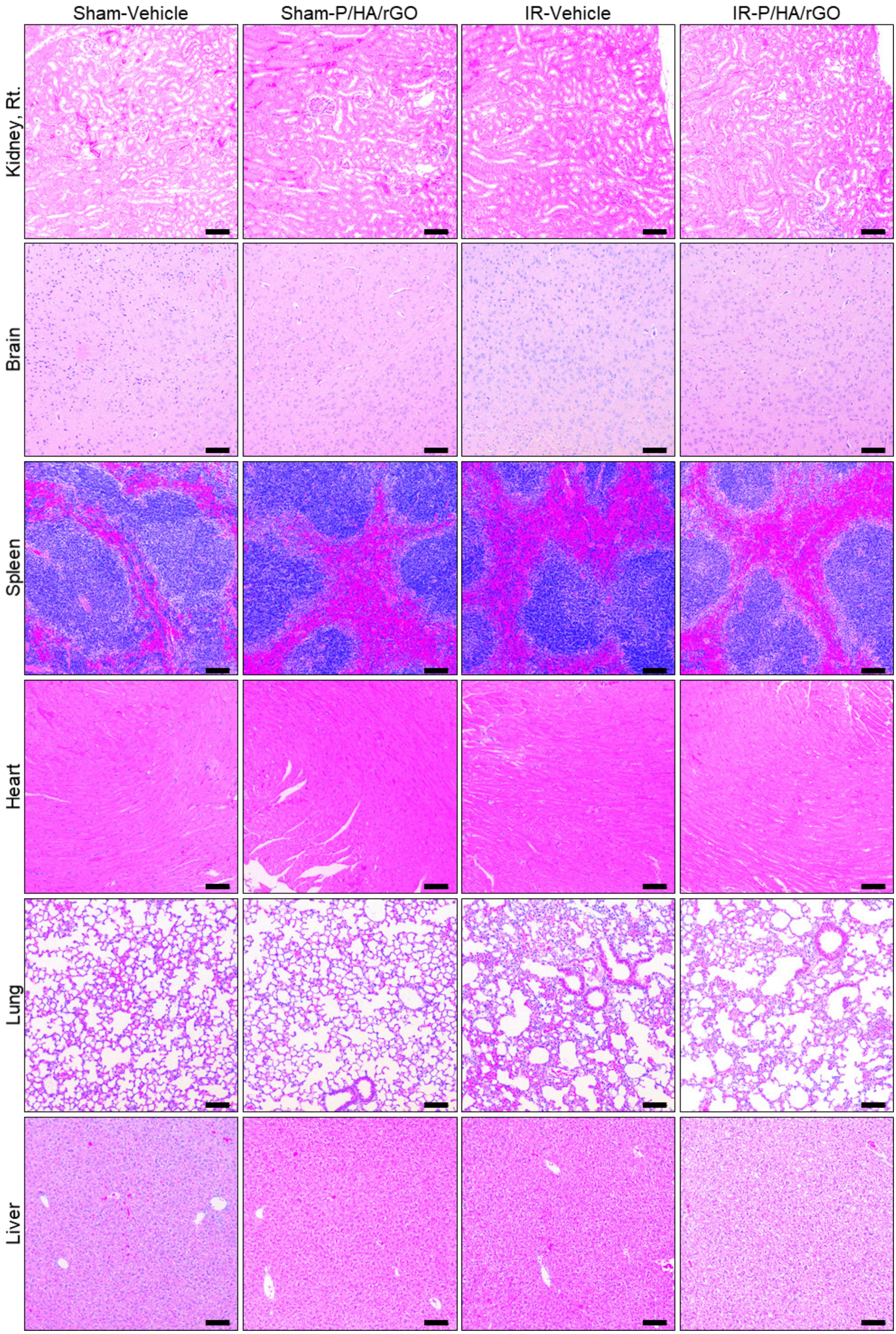

Sham-Vehicle
Sham-P/HA/rGO
IR-Vehicle
IR-P/HA/rGO
Kidney, Rt.
Brain
Spleen
Heart
Lung
Liver

Figure S11. Histological analysis of the various organs following intravenous injection of P/HA/rGO. The representative images of hematoxylin and eosin staining for various organs (brain, heart, right [Rt.] kidney, liver, lung, and spleen) from sham- or IR-operated mice treated with vehicle or P/HA/rGO. Scale bars = 40 μm. IR, ischemia/reperfusion; P/HA/rGO; reduced graphene oxide/hyaluronic acid nanoparticles loaded with paricalcitol

Table S1. Pimary and secondary antibodies employed for immunohistochemistry

Target antigen	Host species	Target species	Vendor	Catalog Number
ACTA2, Cy3-conjugated	Mouse	Mouse	Sigma-Aldrich	C6198
GFP	Chicken	Species independent	Abcam	Ab13970
MHCII	Rat	Mouse	Biolegend	107650
PDGFRβ	Rat	Mouse	Invitrogen	14-1402-81
PECAM-1	Hamster	Mouse	Millipore	MAB1398Z
Podocin	Rabbit	Human, Mouse	Sigma-Aldrich	P0372
TER-119, FITC-conjugated	Rat	Mouse	Biolegend	116206
VEGFR2	Goat	Mouse	R&D systems	AF644
vWF	Sheep	Human, Mouse	Abcam	Ab11713
Anti-Chicken IgG, Alexa 488 conjugated	Donkey	Chicken	Jackson ImmunoResearch	703-545-155
Anti-Goat IgG, Alexa 488 conjugated	Donkey	Goat	Jackson ImmunoResearch	705-545-147
Anti-Hamster IgG, Alexa 647 conjugated	Donkey	Hamster	Jackson ImmunoResearch	127-605-160
Anti-Rabbit IgG, Alexa 594 conjugated	Donkey	Rabbit	Jackson ImmunoResearch	711-585-152
Anti-Rat IgG, Alexa 488 conjugated	Donkey	Rat	Jackson ImmunoResearch	712-545-153
Anti-Sheep IgG, Alexa	Donkey	Sheep	Jackson ImmunoResearch	713-585-147

Table S2. Primer sequences utilized for quantitative real-time PCR (qPCR)

Target gene	Forward	Reverse
Acta2	ACTGGGACGACATGGAAAAG	CATCTCCAGAGTCCAGCACA
Vimentin	CTTGAACGGAAAGTGGAATCCT	GTCAGGCTTGGAAACGTCC
Col4a1	ATTAGCAGGTGTGCGGTTTG	ATTAGCAGGTGTGCGGTTTG
Fn1	ACACGGTTTCCCATTACGCCAT	AATGACCACTGCCAAAGCCCAA
TGFβ1	CTCCCGTGGCTTCTAGTGC	GCCTTAGTTTGGACAGGATCTG
Mmp2	GGACCCCGGTTTCCCTAA	CAGGTTATCAGGGATGGCATTC
Timp2	TTTTGCAATGCAGACGTAGTGA	CCGGAATCCACCTCCTTCTC
Krt7	ACGGCTGCTGAGAATGAGTT	CGTGAAGGGTCTTGAGGAAG
Krt8	GGACATCGAGATCACCACCT	TGAAGCCAGGGCTAGTGAGT
Krt18	CAAGTCTGCCGAAATCAGGGAC	TCCAAGTTGATGTTCTGGTTTT
Krt19	CGGTGGAAGTTTTAGTGGGA	AGTAGGAGGCGAGACGATCA
Tnfα	GGTGCCTATGTCTCAGCCTCTT	GCCATAGAACTGATGAGAGGGAG
Ifng	ATGAACGCTACACACTGCATC	CCATCCTTTTGCCAGTTCCTC
Il1β	AGTTGACGGACCCCAAAAGAT	GGACAGCCCAGGTCAAAGG
Il6	CCACTTCACAAGTCGGAGGCTT	CCAGCTTATCTGTTAGGAGA
GAPDH	TGTGTCCGTCGTGGATCTGA	GATGCCTGCTTCACCACCTT

Table S3. Primary and secondary antibodies employed for immunoblotting

Target antigen	Host species	Target species	Vendor	Catalog Number
ACTA2	Mouse	Human, Mouse, Rat	Sigma-Aldrich	A2547
Vimentin	Rabbit	Human, Mouse, Rat	Cell Signaling	#5741
Collagen IV alpha I	Rabbit	Human, Mouse	Abcam	ab227616
Type I Collagen	Rabbit	Human	Abcam	ab138492
Fibronectin	Rabbit	Human, Mouse	Abcam	ab2413
GAPDH	Mouse	Human, Mouse, Rat	Santa Cruz	sc-32233
MMP-2	Rabbit	Human, Mouse	Cell Signaling	#87809
TIMP2	Rabbit	Human, Mouse	Cell Signaling	#5738
Cytokeratin 7	Rabbit	Human, Mouse, Rat	Abcam	ab181598
Cytokeratin 8	Rabbit	Human, Mouse, Rat	Abcam	ab53280
IGFBP7	Rabbit	Human, Mouse	Cell Signaling	#64563
BAX	Rabbit	Human, Mouse, Rat	Cell Signaling	#2772
Bcl-2	Mouse	Human, Mouse, Rat	Santa Cruz	sc-7382
Cleaved caspase-3	Rabbit	Human, Mouse, Rat	Cell Signaling	#9661
Caspase3	Rabbit	Human, Mouse, Rat	Cell Signaling	#9662
Phospho-SMAD2/SMAD3	Rabbit	Human, Mouse, Rat	Cell Signaling	#8828
SMAD2/3	Rabbit	Human, Mouse, Rat	Cell Signaling	#3102
SMAD4	Rabbit	Human, Mouse, Rat	Cell Signaling	#38454
